# Comprehensive proteomics and platform validation of urinary biomarkers for bladder cancer diagnosis and staging

**DOI:** 10.1186/s12916-023-02813-x

**Published:** 2023-04-05

**Authors:** Kamala Vanarsa, Jessica Castillo, Long Wang, Kyung Hyun Lee, Claudia Pedroza, Yair Lotan, Chandra Mohan

**Affiliations:** 1 Department Biomedical Engineering, University of Houston, 3517 Cullen Blvd., Room 2027, Houston, TX 77204-5060 USA; 2 Department of Urology, Third Xiangya Hospital of Central South University, Changsha, China; 3 Center for Clinical Research and Evidence-Based Medicine, University of Texas Health Science Center at Houston, Houston, TX USA; 4 Department of Urology, UT Southwestern Medical Center, Dallas, TX USA

**Keywords:** Bladder cancer, Proteomics, Biomarkers, Protein biomarkers, Diagnostic biomarkers, Surveillance biomarkers

## Abstract

**Background:**

Bladder cancer (BC) is among the most common cancers diagnosed in men in the USA. The current gold standards for the diagnosis of BC are invasive or lack the sensitivity to correctly identify the disease.

**Methods:**

An aptamer-based screen analyzed the expression of 1317 proteins in BC compared to urology clinic controls. The top hits were subjected to systems biology analyses. Next, 30 urine proteins were ELISA-validated in an independent cohort of 68 subjects. Three of these proteins were next validated in an independent BC cohort of differing ethnicity.

**Results:**

Systems biology analysis implicated molecular functions related to the extracellular matrix, collagen, integrin, heparin, and transmembrane tyrosine kinase signaling in BC susceptibility, with HNF4A and NFKB1 emerging as key molecular regulators. STEM analysis of the dysregulated pathways implicated a functional role for the immune system, complement, and interleukins in BC disease progression. Of 21 urine proteins that discriminated BC from urology clinic controls (UC), urine d-dimer displayed the highest accuracy (0.96) and sensitivity of 97%. Furthermore, 8 urine proteins significantly discriminated MIBC from NMIBC (AUC = 0.75–0.99), with IL-8 and IgA being the best performers. Urine IgA and fibronectin exhibited the highest specificity of 80% at fixed sensitivity for identifying advanced BC.

**Conclusions:**

Given the high sensitivity (97%) of urine d-dimer for BC, it may have a role in the initial diagnosis or detection of cancer recurrence. On the other hand, urine IL-8 and IgA may have the potential in identifying disease progression during patient follow-up. The use of these biomarkers for initial triage could have a significant impact as the current cystoscopy-based diagnostic and surveillance approach is costly and invasive when compared to a simple urine test.

**Supplementary Information:**

The online version contains supplementary material available at 10.1186/s12916-023-02813-x.

## Background

Bladder cancer (BC) is the fourth most common cancer diagnosed in men in the USA [1]. The incidence rate of the disease is four times higher in men than in women and approximately twice as high in White men compared to Black men [1]. It is estimated that 6% of all new cancer diagnoses in men in the year 2022 will be BC [1]. Overall, 81,180 people are expected to be newly diagnosed with BC in 2022, of which 61,700 being male and 19,480 being female [1]. It is also estimated that 17,100 people will die from the disease in 2022 [1]. The risk of developing BC increases with age with the highest risk being in 80-year-old males and females [2]. The 5-year relative survival rate for those with BC is 77% [1]. If the tumor is non-invasive, the 5-year survival increases to 96% [1]. However, 51% of all cases are diagnosed after this occurrence [1].

The current gold standard for the diagnosis of BC is cystoscopy. However, cystoscopy is often associated with complications including pain, urinary tract infection, and hematuria. Urine cytology is also commonly used for the diagnosis and surveillance of BC. This non-invasive method involves the examination of cells collected from a urine specimen. Research has indicated a high specificity of 86%, but it is constrained by a low sensitivity of 48% [3]. There is also subjectiveness when grading urothelial carcinoma on urine samples thus resulting in poor inter-observer variability [3].

The United States Food and Drug Administration (FDA) has approved 6 urinary assays to use in conjunction with cystoscopy for the surveillance and diagnosis of BC. These include BTA stat, BTA TRAK, NMP22 BladderChek Test, NMP22 ELISA, UroVysion, and uCyt [4]. A meta-analysis of NMP-22 BladderChek shows a pooled specificity of 88% and a sensitivity of 56% for BC detection from 19 research studies [5]. The sensitivity of the test was found to steadily increase with higher stages and grades of the disease. An additional meta-analysis of BTA stat identified a pooled specificity of 67% and a sensitivity of 75% in 13 research studies [6]. Similar to NMP22, BTA stat’s sensitivity positively correlates with an increasing grade of BC. Due to false positives and lower specificity values, these tests cannot be used as the sole measure of diagnosis and surveillance. The American Urologic Association guidelines for the evaluation of hematuria and surveillance of bladder cancer do not currently recommend the routine use of urine markers [7, 8].

Given these metrics, there is a need for better biomarkers for BC. Urine biomarkers are promising as a non-invasive test for BC. Urine can be obtained non-invasively, is a readily available biological fluid, and is close to the site of pathology. This allows for repeated tests as deemed necessary for both diagnosis and potential monitoring of disease progression. Urine is also advantageous for potential cost-effective point-of-care tests. Emerging urine point-of-care tests may empower individuals to monitor their health from the comfort of their own homes [9].

As opposed to previous studies in the field examining a handful of proteins selected based on their known properties, here, we report the first and largest use of a comprehensive aptamer-based proteomic screen of urine samples from 42 subjects. This platform has been successfully applied in biomarker screens of several other diseases [10–21]. Additionally, in the present study, we have executed the largest ELISA validation study in BC, interrogating 30 protein biomarkers in an independent cohort consisting of 68 subjects (31 urology clinic controls (UC) and 37 BC (10 Ta, 10 Tis, 10 T1, and 7 T2–T4)). The study has uncovered novel urine protein biomarkers that have not been reported in BC patients before and that out-perform current biomarkers used in clinical practice. The reported urine biomarkers may be useful for the initial diagnosis of BC and possibly for the surveillance of the disease.

## Methods

### Patient cohorts

Inclusion and exclusion criteria: In all cohorts, the included bladder cancer patients were patients in whom the diagnosis was established by cystoscopy and pathology. Subjects with other malignancies were excluded. Urine samples for the initial aptamer-based screen were obtained from the University of Texas Southwestern Medical Center and Bioreclamation (Bioreclamation, RRID:SCR_004728), Westbury, NY. The samples included 15 urology clinic controls (“UC”) and 27 bladder cancer subjects including 5 Ta (non-invasive papillary carcinoma), 4 Tis (flat carcinoma in situ), 9 T1 (tumor spread to connective tissue), 4 T2 (muscle-invasive bladder cancer), 3 T3, and 2 T4 BC. Of these, 18 subjects (stage Ta, Tis, and T1) were classified as non-muscle invasive bladder cancer (NMIBC) while 9 subjects (stage T2–T4) were classified as muscle invasive bladder cancer (MIBC). It should be stressed out that UC does not refer to urothelial carcinoma but for urology clinic controls. Additionally, unless stated otherwise, all bladder cancer subjects included in this study had urothelial cancer. For replication of the findings from the initial proteomic screen, the independent validation cohort for ELISA consisted of samples obtained from the University of Texas Southwestern Medical Center (“UTSW cohort”). These included 31 UC samples and 37 BC samples (10 Ta, 10 Tis, 10 T1, and 7 T2–T4). Of these, 30 subjects (stage Ta, Tis, and T1) were classified as NMIBC while 7 subjects (stage T2–T4) were classified as MIBC. UC samples included patients investigated for hematuria but found not to have any urological cancers. Subject demographics, including age gender and ethnicity, and clinical information pertaining to these samples are detailed in Table 1. Sex as a biological variable: both genders were included in the study. The secondary validation cohort consisted of samples of Chinese ethnicity. These samples were from the Third Xiangya Hospital of the Central South University in Changsha, China, and comprised 91 BC patients and 77 UC patients (Additional file 1: Table S1). Samples in all cohorts were obtained with informed consent. The study was approved by the institutional review boards at the University of Houston, Houston, TX; UTSW, Dallas, TX; and the Third Xiangya Hospital of the Central South University in Changsha, China. In all cases, urine samples were centrifuged, aliquoted, and stored at − 80 °C, and used for the assays without repeated freeze-thaws. Laboratory researchers performing the assays were blinded to the subject groupings.

**Table 1:** Demographic information pertaining to subjects used for the aptamer-based screen

**Variable**	**Category**	**UC** **(*N* = 15)**	**Ta** **(*N* = 5)**	**Tis** **(*N* = 4)**	**T1** **(*N* = 9)**	**T2–T4** **(*N* = 9)**
Age^a^		66.2 ± 4.1	66.6 ± 12.1	68.5 ± 4.7	69.3 ± 10.1	71.9 ± 12.4
Gender, *n* (%)	Male	15 (100.0%)	5 (100.0%)	4 (100.0%)	9 (100%)	9 (100%)
Ethnicity, *n* (%)	Caucasian	6 (40.0%)	4 (80.0%)	4 (100.0%)	5 (55.6%)	5 (55.6%)
	Others	1 (6.6%)	N/A	N/A	N/A	N/A
	Unknown	8 (53.3%)	1 (20.0%)	N/A	4 (44.4%)	4 (44.4%)
**Demographic information pertaining to the ELISA validation cohort (UTSW cohort)**						
**Variable**	**Category**	**UC** **(*N* = 31)**	**Ta** **(*N* = 10)**	**Tis** **(*N* = 10)**	**T1** **(*N* = 10)**	**T2–T4** **(*N* = 7)**
Age^a^		66.2 ± 10.1	71.3 ± 11.9	71.5 ± 6.3	67.4 ± 9.9	74.3 ± 7.9
Gender, *n* (%)	Male	27 (87.1%)	8 (80%)	10 (100%)	8 (80%)	7 (100%)
	Female	4 (12.9%)	2 (20%)	N/A	2 (20%)	N/A
Ethnicity, *n* (%)	Caucasian	23 (74.2%)	8 (80%)	9 (90%)	8 (80%)	6 (85.7%)
	Black	4 (12.9%)	N/A	N/A	1 (10%)	1 (10%)
	Asian	2 (6.5%)	1 (10%)	N/A	1 (10%)	N/A
	Hispanic	N/A	1 (10%)	N/A	N/A	N/A
	Others	1 (3.2%)	N/A	N/A	N/A	N/A
	Unknown	1 (3.2%)	N/A	1 (10%)	N/A	N/A

Top: Demographic information pertaining to the 42 subjects included in the aptamer-based screen. NMIBC comprised Ta, Tis, and T1 subjects. MIBC comprised T2–T4 BC. Subjects in the UC category comprised of BPH (*n* = 1), elevated PSA (*n* = 1), erectile dysfunction (*n* = 2), HLD (*n* = 2), allergic rhinitis (*n* = 2), hypothyroidism (*n* = 2), dementia (*n* = 1), insomnia (*n* = 1), impotence (*n* = 1), osteopenia (*n* = 1), thrombocytopenia (*n* = 1), hemangioma (*n* = 1), glaucoma (*n* = 1), and gout (*n* = 1)

Bottom: Demographic information pertaining to the 68 subjects used for the independent ELISA validation cohort UTSW Cohort. NMIBC comprised Ta, Tis, and T1 subjects. MIBC comprised T2–T4 BC. UC subjects comprised patients with various urological conditions including bladder diverticulum (*n* = 1), bladder stones (*n* = 1), BPH (*n* = 7), cystocele (*n* = 1), elevated PSA (*n* = 2), erectile dysfunction (*n* = 3), hematuria (*n* = 3), UTI (*n* = 1), hydrocele (*n* = 1), incomplete bladder emptying (*n* = 1), impotence (*n* = 1), kidney stones (*n* = 3), nocturia (*n* = 2), prostate cancer (*n* = 1), and stress incontinence (*n* = 1)

^a^Age displayed as mean ± standard deviation

### Aptamer-based targeted proteomic screen of BC urine

The samples for the aptamer-based screen consisted of 15 urology controls and 27 bladder cancer subjects (BC Ta = 5, BC Tis = 4, BC T1 = 9, BC T2 = 4, BC T3 = 3, BC T4 = 2). These urine samples were screened using an aptamer-based screening platform (“SOMAScan”) manufactured by Somalogic, as detailed previously [20, 22]. In short, the samples were added to the aptamer-coated beads. SOMAmer-protein binding then occurs. Following this, the unbound proteins are washed off. The remaining bound proteins are biotinylated. SOMAmer-protein complexes are next photocleaved from the beads with UV light. Incubation in a buffer with a polyanionic competitor disrupts non-specific interactions. The SOMAmer-proteins are then recaptured on a second streptavidin-coated bead. Next, the SOMAmer reagents are released from the beads in a denaturing buffer. The released SOMAmers are then hybridized onto a DNA microarray and quantified by the relative fluorescence unit for each protein.

### Cross-sectional ELISA validation of urine protein biomarkers

Altogether, 34 proteins were initially selected for ELISA validation based on the aptamer-based screening. Commercially available ELISA kits were purchased, and preliminary testing was conducted. The protein, ELISA manufacturer, optimal urine sample dilution, reason for selection, and outcome of ELISA testing are listed in Additional file 1: Table S2. After preliminary testing, 30 protein biomarkers were assayed in an independent (UTSW) cohort which consisted of 31 UC samples and 37 BC samples (10 Ta, 10 Tis, 10 T1, and 7 T2–T4). The absolute levels of urine proteins were creatinine normalized. Secondary validation was completed for 3 protein biomarkers using an independent cohort comprising 91 BC patients and 77 patients of Chinese ethnicity. The demographic information and clinical information pertaining to the 168 subjects whose urine samples were used for the second ELISA validation are displayed in Additional file 1: Table S1. In this cohort, most patients classified as BC were diagnosed to have bladder cancer except for 7 patients with urothelial cancers (including ureteric cancer). After validation, the absolute levels of urine proteins were normalized by creatinine. The ELISA assay protocols are detailed as has been shown before [20].

### d-dimer assay used in the Chinese cohort

As opposed to the ELISA used to assay d-dimer in the other cohorts, an agglutination assay was used to assay d-dimer in the Chinese cohort. A d-dimer detection kit (latex immunity ratio turbidity method) from SEKISUI was used to quantitatively detect the concentration of d-dimer in the Chinese cohort. Briefly, the d-dimer in the sample reacts with the monoclonal antibody to the mouse anti-human d-dimer. This causes agglutination and increased turbidity. Following this, the concentration of d-dimer was determined by measuring the variation of turbidity with a spectrophotometer.

### Data analysis of the aptamer-based screening and ELISA results

Aptamer-based screening data was subjected to hybridization, median normalization, and creatinine normalization as detailed previously [20]. Further data analysis was completed in R version 1.4.1103 with packages readxl, readr, qvalue, and stats. A non-parametric Mann–Whitney *U*-test was used to identify the proteins that were significantly up- or downregulated among the subject groups. Statistical *p*-values were computed for each biomarker. To address multiple testing correction, *q*-values were calculated to adjust for the false discovery rate. Fold change values were also computed to determine the ratio of protein expression from diseased to control subjects (BC/UC) and MIBC to NMIBC subjects (T2–T4 vs Ta, Tis, T1). ROC analyses including AUC, cutoff, sensitivity, and specificity values were computed with easyROC version 1.3.1 [23]. The biomarker ELISA data was plotted and analyzed using GraphPad Prism 5 (GraphPad Prism, RRID:SCR_002798). Group comparisons were analyzed using either a non-parametric Mann–Whitney *U*-test or a Kruskal–Wallis test with Dunn’s multiple comparison. Statistical *p*-values from analyses were computed.

### Gene Ontology and KEGG functional enrichment analysis

Gene Ontology (GO) and KEGG (KEGG, RRID:SCR_012773) functional enrichment analysis was completed using the Database for Annotation, Visualization, and Integrated Discovery (DAVID) version 6.8 8 (DAVID, RRID: SCR_001881). The top 330 proteins with a Mann–Whitney *p*-value < 0.05 in the aptamer-based screen (BC versus UC) were used for analysis. The top 10 biological processes, molecular functions, and KEGG pathways were plotted using R. The packages used include readxl and ggplot2 (ggplot2, RRID:SCR_014601). The size of the dots represents the count/hit number of genes belonging to the annotation term, and the color of the dots represent − log_10_FDR value.

### Protein–protein interaction networks and regulatory networks

Protein–protein interaction networks for the top 330 proteins in the aptamer-based screen (BC vs UC, Mann–Whitney *p*-value < 0.05) were created using Cytoscape version 3.9.0 (Cytoscape, RRID:SCR_003032) using the stringApp. MCODE clustering was preformed to discern highly interconnected nodes in the network. The top 3 clusters are plotted with their associated Reactome pathways. The top transcription factor and signaling molecule regulator were identified for the top 330 proteins in the aptamer-based screening (BC vs UC, Mann–Whitney *p*-value < 0.05) using the iRegulon plugin available through Cytoscape. The color of each node corresponds to the fold change. Nodes with a fold change of less than 1 range in color from blue to purple while those with a fold change greater than 1 range from pink to red, when comparing BC to UC.

### Volcano plot, principal component analysis (PCA), and correlation plot

The volcano, PCA, and correlation plots were created in R using the readr, readxl, gplots, ggplot2, ggplot.multistats, scatterplot3d, Hmisc, data.table, and corrplot packages. All 1317 proteins are represented in the volcano plot. The data was log-transformed, and a Mann–Whitney *U*-test (BC vs UC) was used to generate statistical *p*-values. A 2D PCA plot was generated for the top 119 proteins (BC vs UC, Mann–Whitney *q*-value < 0.05) where the two first principal components are plotted. Subject groups are differentiated by color and/or shape. A correlation plot for the top 50 proteins was also generated (Mann–Whitney *p*-value < 0.05, ordered by fold change, comparing BC to UC). Correlation coefficients for all possible protein pairs were computed using Pearson’s correlation coefficients.

### Heatmap analysis

Heatmaps were generated from the aptamer-based screening assay in order to cluster proteins with similar expression profiles together. Proteins significantly elevated in BC when compared to urology controls (*p*-value < 0.05 and a fold change > 2) were analyzed. Hierarchical clustering was performed in R. Each row corresponds to the creatinine-normalized protein level measured, and each column represents a sample (UC = 15, BC Ta = 5, BC Tis = 4, BC T1 = 9, BC T2 = 4, BC T3 = 3, BC T4 = 2). Proteins that are above the mean value for each biomarker are shaded yellow. Proteins comparable to the mean are shaded black. Those below the mean are shaded blue.

### Random forest analysis

Random forest analysis, a supervised machine learning algorithm, was conducted for the purpose of identifying the relative importance of biomarker candidates in disease discrimination. The randomForest R package and the top 93 proteins identified from the aptamer-based screen (BC vs UC, Mann–Whitney *p* < 0.05 and FC > 2) were used for analysis. The importance of each biomarker was measured using the GINI coefficient. The resulting top 10 potential urine biomarkers identified by random forest classification are plotted.

### Bayesian network analysis

Bayesian network analysis was executed with the BayesiaLab software. This method uses probability distributions to represent the inter-dependencies between all variables in a model and how they relate to one another. The dataset comprised 68 subjects including 31 UC and 37 BC (10 Ta, 10 Tis, 10 T1, 7 T2–T4) subjects. The urine levels of 21 protein biomarkers, features (age, gender, ethnicity), and disease status (BC vs UC) were examined. The network was constructed in an unsupervised manner with the EQ algorithm. The size of each node was determined using node force and is proportional to its impact on the other nodes in the network. The arcs that interconnect the nodes were determined with Pearson’s correlation coefficient. The interconnections between nodes represent the dependencies among the variables including the correlation coefficient between nodes. The thickness of the arcs is proportional to Pearson’s correlation coefficient.

### STEM analysis

Short Time-series Expression Miner (STEM) version 1.3.13 was utilized for clustering, comparing, and visualizing protein expression data from the aptamer-based screen across bladder cancer (BC Ta-Tis = 9, BC T1 = 9, BC T2–T4 = 9) and urology controls (UC = 15). Protein expression among the top 330 proteins (BC vs UC, Mann–Whitney *p*-value < 0.05) were averaged across the subject groups analyzed. The number in the upper left-hand corner of each box is representative of the number of genes in each cluster. The number in the lower left-hand corner is the *p*-value significance of the number of proteins assigned to the cluster versus what was expected based on permutation testing. Protein expression profiles through UC and progressive BC stages, Ta-Tis, T1, and T2–T4 are plotted. Statistically significant profiles that are similar form a cluster and are shaded the same color. A total of 50 cluster patterns were generated by STEM analysis, of which only the statistically significant clusters that exhibited a progressive increase or decrease of urine biomarkers across BC stages are plotted. The associated Gene Ontology (GO) enrichment analysis for profiles of proteins with the same expression patterns was determined. The Reactome pathways associated with each significant cluster were identified through Cytoscape and the functional enrichment tool.

### Multi-biomarker panels after adjusting for age, gender, and ethnicity

The protein level of each protein assayed by ELISA and the age of patients were standardized to have a mean of zero and a standard deviation of one, after applying log_2_ transformation. The proteins that best discriminate classes of subjects (BC vs UC and MIBC vs NMIBC) were identified using the predictive projection feature selection technique executed with the projpred package in R (version 4.0.3.) [24–26]. Model selection was performed using a model with the best predictive power (a reference model) in order to find a model with a smaller number of proteins. The smaller model should maintain comparable prediction performance when compared to the reference model (predictive projection). The selection process consisted of two main components. During the first step, a Bayesian regularized logistic regression model with horseshoe prior [27, 28] was fitted as a reference model. During the second step, a projected submodel with at most 5 proteins that minimized the Kullback–Leibler divergence from the posterior distribution of the reference model to that of the projected model was searched for. The selected submodel was found to exhibit similar predictive performance determined by the mean log predictive density and the mean square error. Both of the performance metrics in addition to the area under the curve and prediction accuracy were assessed through leave-one-out cross-validation in an effort to bypass potential problems resulting from overfitting [29]. The selected proteins of one model and its performance metrics were compared to those of the counterpart model with adjustments for age, ethnicity, and gender to account for potential confounding factors from these variables.

## Results

### Aptamer-based targeted proteomic screen of BC urine

An overview of the study flow is depicted in Additional file 1: Fig. S1. A comprehensive aptamer-based screen of urine samples from 42 human subjects was executed to interrogate the levels of 1317 proteins, as detailed in the “Methods” section. This study group included 27 bladder cancer subjects and 15 UC (Table 1). A non-parametric Mann–Whitney *U*-test was used to identify proteins that were significantly up- or downregulated among BC vs UC, resulting in 330 proteins. After multiple testing correction, 119 of these 330 proteins had a *q*-value < 0.05. The 330 proteins found to be statistically significantly different (both up- and downregulated proteins) in BC were subjected to functional pathway enrichment.

**Fig. 1:**
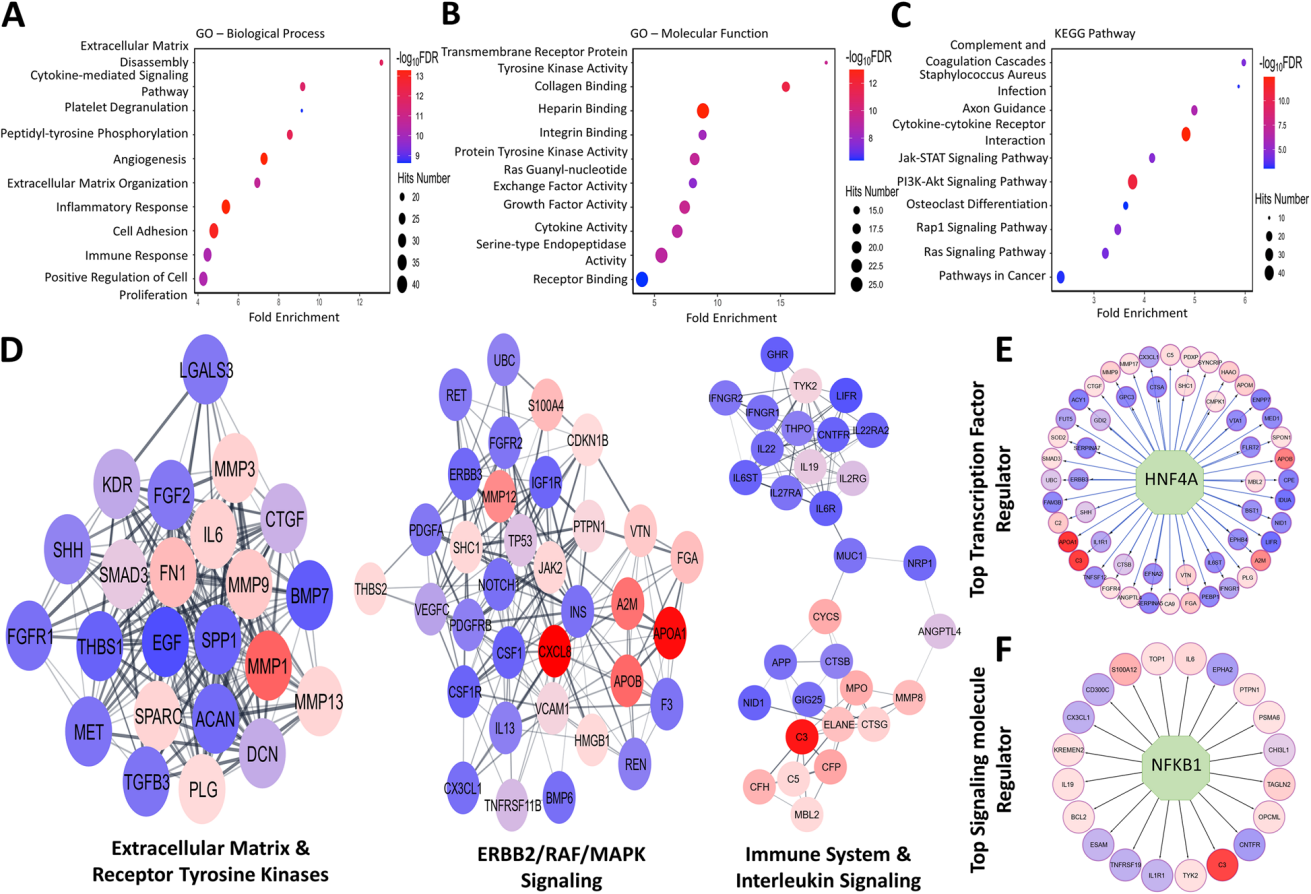
Functional pathway enrichment analysis of proteins dysregulated in bladder cancer urine using Gene Ontology, KEGG pathway, and protein–protein interaction networks. All 330 proteins with a Mann–Whitney *p*-value < 0.05 (BC versus UC) in the aptamer-based screen were used for functional pathway enrichment. **A**–**C** The top 10 Gene Ontology biological process, molecular functions, and KEGG pathways obtained through GO are plotted respectively based on *p*-value significance in the order of fold enrichment. The size of the dots represents the count/hit number of genes belonging to the annotation term, and the color of the dots is representative of − log_10_FDR value. **D** Protein–protein interaction networks for the top 330 proteins (BC vs UC, Mann–Whitney *p*-value < 0.05) were created using the Cytoscape stringApp. MCODE clustering was preformed to find highly interconnected nodes in the network. The top 3 clusters are plotted with their associated Reactome pathways. The color of each node corresponds to the fold change. Nodes with a fold change of less than 1 (reduced in BC) range in color from blue to purple while those with a fold change greater than 1 (increased in BC) range from pink to red. **E** The top transcription factor regulator regulating the differentially expressed proteins was identified using the iRegulon plugin available for Cytoscape. Nodes with a fold change of less than 1 range in color from blue to purple while those with a fold change greater than 1 range from pink to red

Functional pathway enrichment was performed using Gene Ontology analysis. The top 10 Gene Ontology biological processes identified from the differentially expressed urine proteins in BC can be found in Fig. 1A. Extracellular matrix organization, cytokine signaling, inflammatory response, and angiogenesis were identified as some of the most significant biological processes associated with the dysregulated proteins in BC. The top Gene Ontology molecular functions that were enriched included binding to collagen, integrin, heparin, and transmembrane tyrosine kinase receptors (Fig. 1B). KEGG pathway analysis was next executed for these 330 proteins to identify implicated pathways (Fig. 1C). Cytokine-cytokine receptor interaction and PI3K-AKT signaling pathways encompassed the largest number of proteins under these annotation terms.

Following this, protein–protein interaction networks were created with Cytoscape using the 330 differentially expressed proteins in BC to determine their interactions with one another. MCODE clustering was performed to identify highly interconnected clusters (Fig. 1D). Node color is representative of fold change, with downregulated proteins in BC urine shaded blue and upregulated proteins in BC urine shaded red. Reactome pathways enriched among the first cluster include extracellular matrix and receptor tyrosine kinases. The second cluster encompasses ERBB2/RAF/MAPK signaling pathways, while the third cluster is enriched with the immune system and interleukin signaling pathways. HNF4A was identified as the top-most transcription factor controlling the differentially expressed proteins in BC (Fig. 1E), while NFKB1 was singled out as the topmost signaling molecule regulating these proteins (Fig. 1F), as determined using the iRegulon plugin for Cytoscape.

**Fig. 2:**
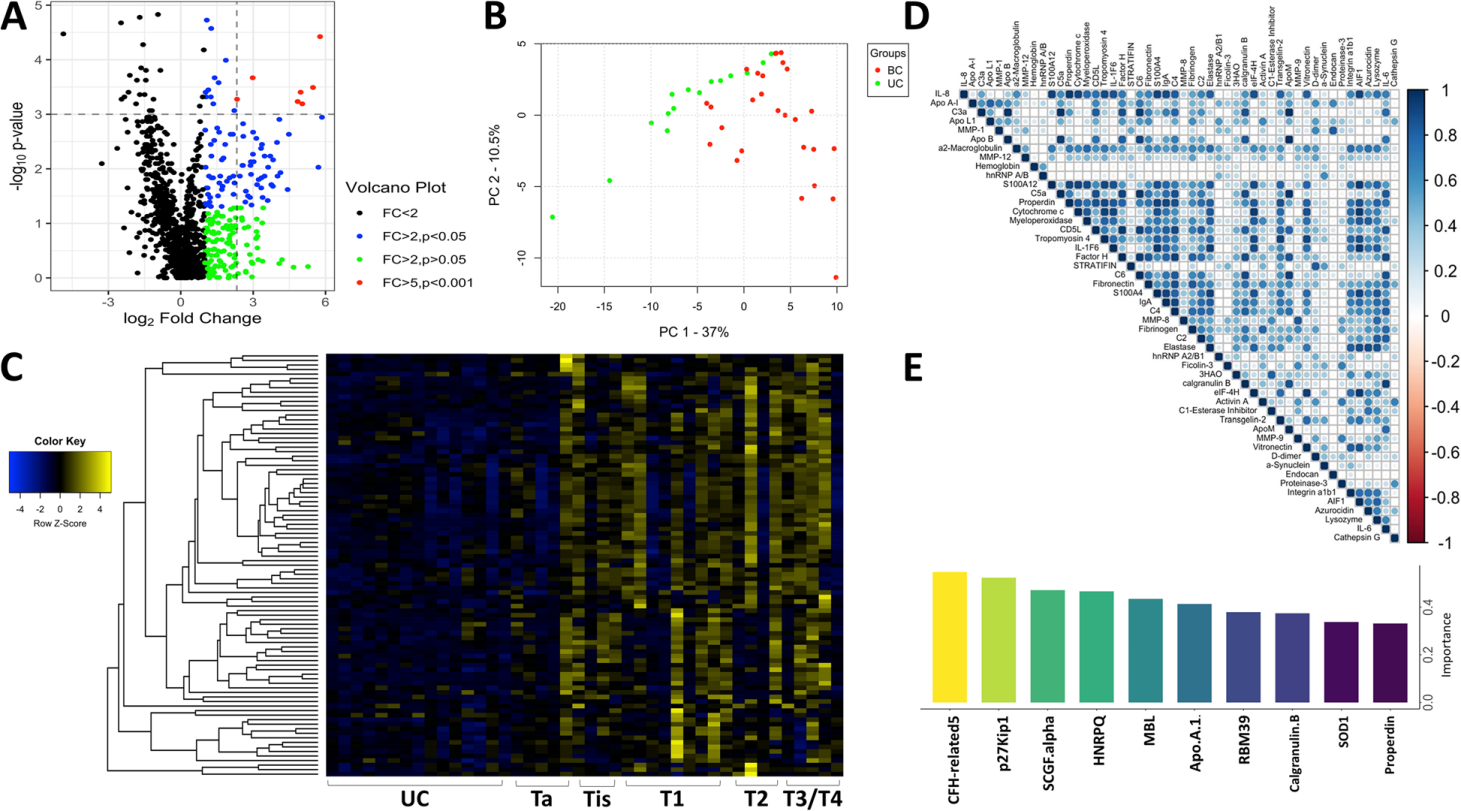
Aptamer-based proteomic screening of bladder cancer urine uncovers several clusters of up- and downregulated proteins. **A** A volcano plot representation of the results of the aptamer-based screening of 1317 proteins analyzed in 42 urine samples (15 UC, BC (Ta-Tis) = 9, BC (T1) = 9, BC (T2–T4) = 9). Data was log-transformed and analyzed as detailed in the “Methods” section. Of the 330 proteins that were differentially expressed between the groups, 93 proteins were elevated at fold change > 2 in BC when compared to UC. Each dot represents one of the 1317 proteins. The *x*-axis plots the log_2_ transform of the fold change. The *y*-axis displays the − log_10_ transform of the *p*-value. **B** A 2D PCA plot of all subjects, using the 119 proteins that were differentially expressed after multiple testing corrections (BC vs UC, Mann–Whitney *q*-value < 0.05). Bladder cancer is represented by a red circle while urology control is represented by a green circle. The first three principal components are displayed on each axis of the plot. **C** A heatmap representation of the results of the aptamer-based screen displaying the top 93 proteins (BC vs UC, Mann–Whitney *p*-value < 0.05, fold change > 2) elevated in BC urine. Hierarchical clustering was performed. Each row corresponds to the creatinine-normalized protein level measured, and each column represents a patient sample (UC = 15, BC Ta = 5, BC Tis = 4, BC T1 = 9, BC T2 = 4, BC T3 = 3, BC T4 = 2). Proteins that are above the mean value for each biomarker and shaded yellow. Those below the mean are shaded blue. Proteins comparable to the mean are shaded black. **D** Correlation plot displaying the expression profiles of the upregulated proteins in BC across the entire cohort. Pearson’s and Spearman’s correlation coefficients were determined for each pair. The proteins were ordered based on hierarchical clustering. Each circle represents the correlation for a protein pair. Blue corresponds to a positive correlation while red corresponds to a negative correlation. **E** Random forest analysis using the top 93 proteins (BC vs UC, Mann–Whitney *p*-value < 0.05, fold change > 2) identified the 10 most discriminatory urine proteins with the greatest impact on distinguishing BC subjects from urology controls. These 10 proteins are ordered by their GINI coefficient (importance in discrimination)

Of the 1317 proteins assayed in the aptamer-based screen, 93 urine proteins were found to be elevated in BC vs UC (Mann–Whitney *p*-value < 0.05) at a fold change of > 2, as depicted by the volcano plot in Fig. 2A. Of these 93 proteins, 7 were found to be significantly elevated with a Mann–Whitney *p*-value < 0.001 and a fold change of > 5 (represented as red dots in Fig. 2A). The top 119 proteins (BC vs UC, *q*-value < 0.05) were used as input for principal component analysis (PCA), an unsupervised machine learning algorithm, which successfully discriminated BC patients from the urology clinic controls (Fig. 2B). The first two principal components are displayed, with BC and UC samples being represented by a red and green circle, respectively. Additional file 1: Fig. S2A. and S2B display additional PCA plots for all expressed proteins and all 330 differentially expressed proteins, respectively.

A heatmap clustering of the 93 proteins significantly elevated in BC is shown in Fig. 2C. Proteins with upregulated expression are colored yellow while those downregulated are colored blue. To help select proteins for further ELISA validation, the expression profiles of the top 50 upregulated proteins (based on fold change) were next clustered using a correlation matrix (Fig. 2D). A correlation plot of the 93 significantly elevated proteins in BC was also generated (Additional file 1: Fig. S3). Several of these urine proteins were highly correlated with each other, as marked by distinct subclusters of urine proteins that became evident. As an independent approach to identify the most discriminatory proteins, a machine learning approach was used. Specifically, random forest analysis (RFA) of the top 93 proteins (BC vs UC, Mann–Whitney *p*-value < 0.05, fold change > 2) identified the 10 most discriminatory urine proteins with the greatest impact on distinguishing BC from UC (Fig. 2E), ordered by their importance as represented by their mean decrease in Gini coefficient. Both the correlation clusters (Fig. 2D) and the RFA (Fig. 2E) were used to select proteins for subsequent ELISA validation.

### Urine biomarkers for distinguishing bladder cancer stages, based on the aptamer-based screen

**Fig. 3:**
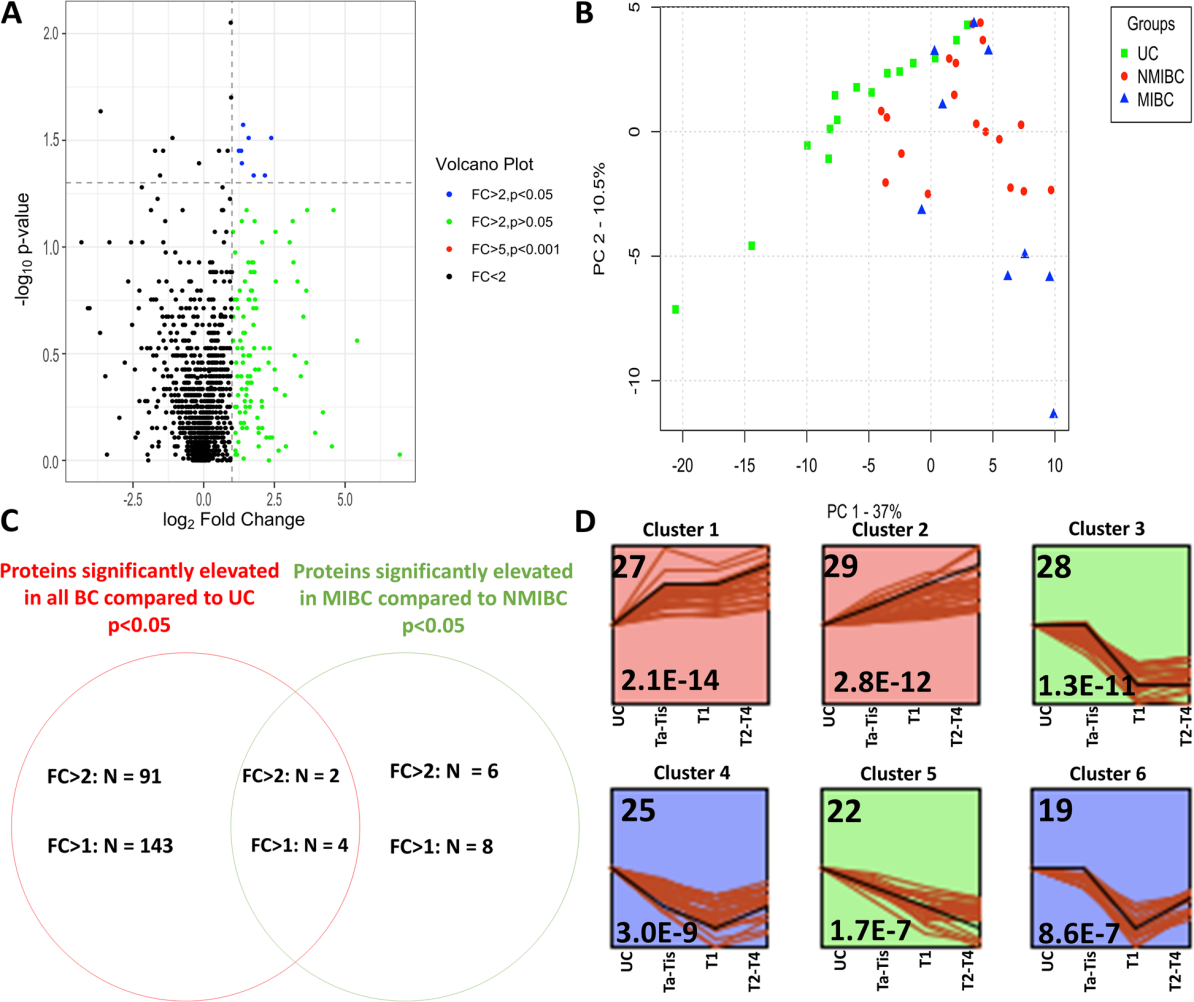
Urine proteins that discriminate BC stages, based on the aptamer-based proteomic screen. **A** A volcano plot representation of the results of the aptamer-based screening of 1317 proteins analyzed in 27 urine samples, comparing disease stages (NMIBC = 18, MIBC = 9). Data was log-transformed and analyzed as detailed in the “Methods” section. Eight proteins were found to be elevated (Mann–Whitney *p*-value < 0.05, fold change > 2) in MIBC when compared to NMIBC. Each dot represents one of the 1317 proteins. The *x*-axis shows the log_2_ transform of the fold change. The *y*-axis displays the − log_10_ transform of the *p*-value. **B** A 2D PCA analysis of all subjects using the top 119 urine proteins (BC vs UC, Mann–Whitney *q*-value < 0.05). NMIBC are represented by a red circle while MIBC is represented by a blue triangle. Urology control is represented by a green square. The first three principal components are displayed on each axis of the plot. **C** A Venn diagram comparison of the number of proteins significantly elevated in bladder cancer versus urology control compared to proteins significantly elevated in MIBC compared to NMIBC urine. The data for two different fold change cutoffs are included. **D** Short Time-series Expression Miner (STEM) analysis was completed for the top 330 proteins (BC vs UC, Mann–Whitney *p*-value < 0.05). The number in the upper left-hand corner of each box is the number of proteins in each cluster. The number in the lower left-hand corner is the *p*-value significance of the number of proteins assigned to the cluster versus what was expected based on permutation testing. The protein expression through UC, and progressive BC stages, Ta-Tis, T1, and T2–T4, is plotted. Statistically significant profiles that are similar form a cluster of profiles and are shaded the same color. A total of 50 profiles or clusters (each representing a different pattern) were generated by STEM analysis, of which only the statistically significant clusters that exhibited a progressive increase (clusters 1 and 2) or decrease (clusters 3–6) of urine biomarkers across BC stages are plotted

Next, we evaluated if the urine proteins identified in the aptamer screen were able to distinguish earlier BC stages from later stages. Urine protein levels in NMIBC were compared to the corresponding levels in MIBC, as summarized by the volcano plot in Fig. 3A, which plots statistical significance (*y*-axis) versus biological significance (*x*-axis). With this comparison, 8 urine proteins were found to be elevated in MIBC stages compared to NMIBC (MIBC vs NMIBC, Mann–Whitney *p*-value < 0.05, fold change > 2), as represented by the blue dots. Principal component analysis (PCA) demonstrated that the differentially expressed proteins in BC were successful in discriminating BC patients with more advanced disease (MIBC), from the urology clinic controls (Fig. 3B). Additional PCA plots display all three subject groups (UC, NMIBC, and MIBC) for all expressed proteins and all 330 differentially expressed proteins (Additional file 1: Fig. S2C and S2D).

A Venn diagram representation of the significantly elevated proteins identified through the aptamer-based screen is shown in Fig. 3C. More proteins were found to be significantly elevated in BC compared to UC (BC vs UC, Mann–Whitney *p*-value < 0.05) with 93 upregulated at a fold change of 2, and 147 upregulated at a fold change of 1. In contrast, 8 proteins were identified as being significantly elevated with a fold change of 2 or greater, and 12 proteins obtained a fold change greater than 1 in MIBC compared to NMIBC (Mann–Whitney *p*-value < 0.05). Four urine proteins overlapped between these two comparisons with a fold change greater than 1. These four overlapping proteins included urinary Elastase, S100A12, p53, and Kallikrein 6.

**Fig. 4:**
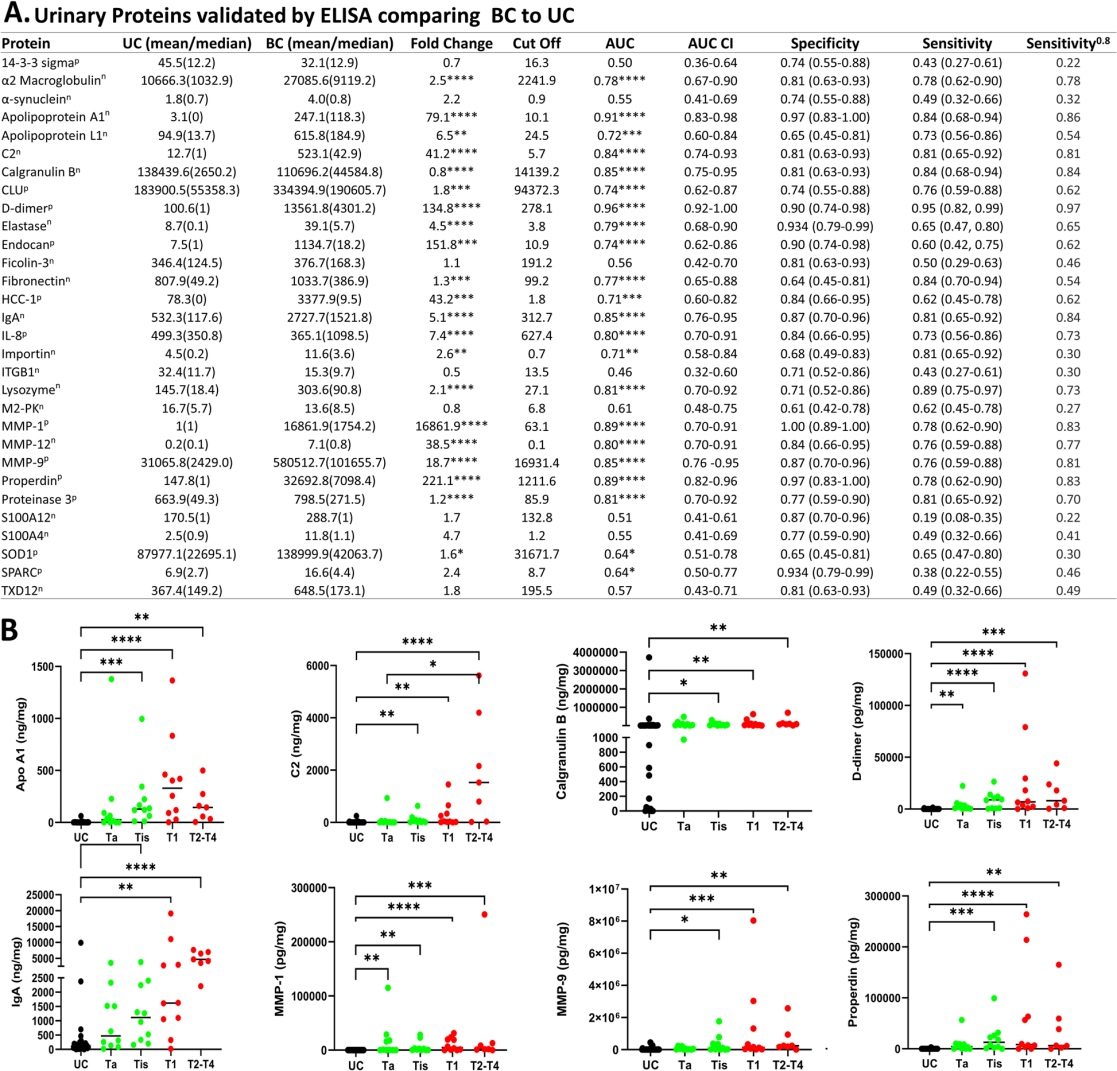
The ability of 30 ELISA-validated urine proteins to discriminate bladder cancer patients from urology clinic controls. **A** Thirty urinary proteins were assayed in bladder cancer and UC by ELISA, using the UTSW cohort, comprising 31 UC, 10 Ta, 10 Tis, 10 T1, and 7 T2-T4 BC patients. The ELISA results for BC vs UC are displayed. The urology clinic controls (UC) comprise urology clinic control patients without urological cancers, as detailed in the "Methods" section. Biomarker protein units (normalized to creatinine) are as follows: *n* = ng/mg, *p* = pg/mg. CI: confidence interval. **A** Delong CI for AUC and a Clopper-Pearson CI for sensitivity and specificity is displayed. Sensitivity.^0.8^ depicts the sensitivity at a fixed specificity value of 0.8. Indicated are the statistical significance *p*-values *p < 0.05, **p < 0.01, ***p < 0.001, ****p < 0.0001, comparing BC to UC. **B** The dot plots depict the 8 proteins with the highest ROC AUC accuracy values for discriminating BC vs UC. Creatinine normalized urine protein levels are plotted in contrasting colors representing UC, BC Ta, Tis, and T1–T4 stages. Statistical analyses of plots were carried out using a Kruskal-Walls test with Dunn’s multiple comparison post hoc test. The asterisks indicate the level of significance between the groups: *p < 0.05, **p < 0.01, ***p < 0.001, ****p < 0.0001

Next, to identify urine proteins and functional pathways that progressively increased or decreased with the worsening BC stage, Short Time-series Expression Miner (STEM) analysis was executed. This analysis identified 2 distinct clusters of proteins that progressively increased with BC stage (clusters 1 and 2; left 2 boxes in Fig. 3D) and 4 clusters of proteins that decreased with worsening BC stage (rightmost 4 boxes in Fig. 3D). The mean expression profiles of the proteins assigned to each plot are represented by the black line within each box. Functional pathway enrichment using Reactome indicated that the proteins in cluster 1 (27 proteins) and those in cluster 2 (29 proteins) were significantly enriched with pathways related to the immune system, complement cascade, and interleukin signaling. Reactome or KEGG functional pathways identified by the proteins in clusters 3–6 included MAPK signaling, cytokine-cytokine receptor interaction, Rap 1 signaling, interleukin signaling, EPH-ephrin mediated repulsion of cells, and signaling by receptor tyrosine kinases.

### Validation of urine protein biomarkers in BC urine using an additional assay platform, ELISA, using an independent patient cohort

Based on the correlation clustering (Fig. 2D) and random forest analysis (Fig. 2E) of the urine proteins identified using the aptamer-based screen, 34 proteins were selected for ELISA validation, which represents a different platform than the one used for the initial screen. The selected proteins, ELISA manufacturer, urine dilution, reason for protein selection, and outcome of the ELISA are listed in Additional file 1: Table S2. Of the 34 proteins selected, 30 proteins were advanced forward for validation in the first independent cohort based on preliminary ELISA results.

These 30 proteins were assayed by ELISA in a cohort of 68 urine samples, drawn from 31 UC, and 37 BC patients, comprising 10 Ta, 10 Tis, 10 T1, and 7 T2–T4 patients, referred to in this manuscript as the UTSW cohort (Table 1). The BC vs UC comparison of the ELISA results is detailed in Fig. 4A which includes all of the data for the 30 proteins. This data was creatinine normalized. A box plot view depicting the expression profile of each protein is displayed in Additional file 1: Fig. S4. The eight urine proteins with the highest area under the curve (AUC) for discriminating BC from UC include Apo A1, complement C2, Calgranulin B, d-dimer, IgA, MMP-1, MMP-9, and Properdin. Their AUC values ranged from 0.84 to 0.96. Figure 4B displays the dot plot views for these eight urine proteins. Of all ELISA-tested proteins, urine d-dimer was best at discriminating BC from UC (AUC = 0.96, sensitivity = 95%; specificity = 90%), with urine properdin and MMP-1 being close behind. As a sensitivity analysis, a bootstrap logistic regression model was used to derive optimism-corrected performance metrics and to ascertain the robustness of each urine protein for distinguishing BC from urology control. This method, which is more accurate for small sample sizes, yielded similar results, as listed in Additional file 1: Table S3.

**Fig. 5:**
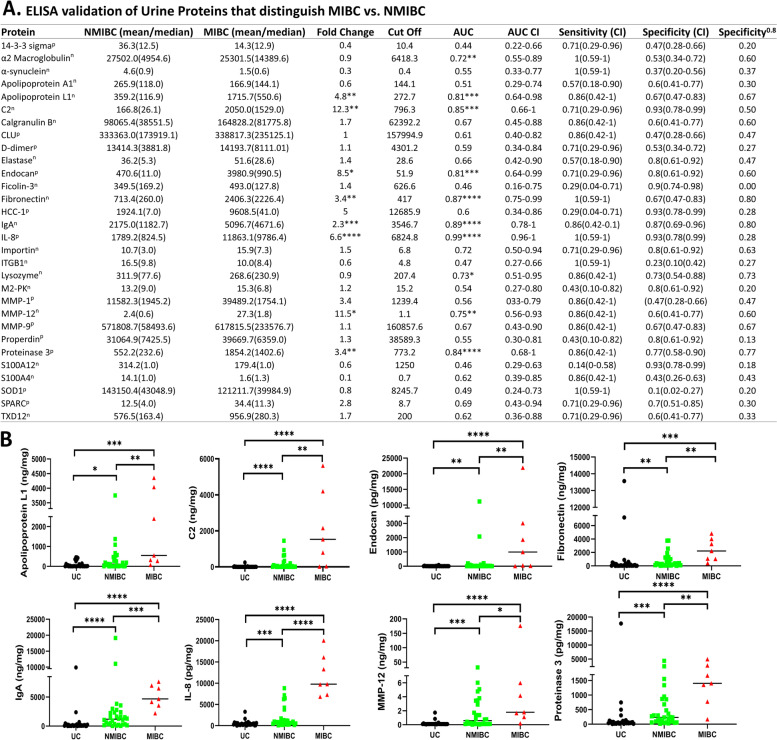
The ability of 30 ELISA-validated urine proteins to discriminate bladder cancer patients by their disease stage. **A** Thirty urinary proteins were assayed by ELISA, using the UTSW cohort, comprised of 7 MIBC and 30 NMIBC subjects. The ELISA results for MIBC vs NMIBC are displayed. Biomarker protein units (normalized to creatinine) are as follows: *n* = ng/mg, *p* = pg/mg. Specificity.^0.8^ depicts the specificity at a fixed sensitivity of 0.8. Indicated are the statistical significance *p*-values as determined by a Mann-Whitney *U*-test *p < 0.05; **p < 0.01; ***p < 0.001; ****p < 0.0001. **B** The dot plots depict the 8 proteins with the highest AUC value for MIBC vs NMIBC comparison. Tested samples include 30 NMIBC (10 Ta, 10 Tis, 10 T1) and 7 MIBC (T2–T4). Creatine-normalized urine protein levels are shown in different colors representing UC, NMIBC, and MIBC. The asterisks indicate the level of significance between the groups: *p < 0.05, **p < 0.01, ***p < 0.001, ****p < 0.0001

Besides comparing the different BC groups to UC, MIBC (T2–T4) was also compared to NMIBC (Ta, Tis, and T1). The results of the 30 ELISAs for the MIBC vs NMIBC stage comparisons are detailed in Fig. 5A and Additional file 1: Fig. S5. This data was creatinine-normalized. Figure 5B displays the dot plot view for the eight urine proteins with the highest AUC values for discriminating MIBC from NMIBC. These included urine Apolipoprotein L1, complement C2, Endocan, Fibronectin, IgA, IL-8, MMP-12, and Proteinase 3. Their AUC values ranged from 0.75 to 0.99. Urine IL-8 was best at discriminating these two groups (AUC = 0.99, sensitivity = 100%; specificity = 93%). Urine IgA also outperformed other markers, with an AUC of 89%, a sensitivity of 86%, and a specificity of 87%. Of particular note, urine IgA exhibited the highest specificity of 80% for MIBC, at 80% sensitivity, outperforming IL-8. As a sensitivity analysis, a bootstrap logistic regression model was used to derive optimism-corrected performance metrics and to ascertain the robustness of each urine protein for distinguishing MIBC from NMIBC. This method yielded similar results, as listed in Additional file 1: Table S4.

### Multi-marker panels and Bayesian network analysis of urine protein biomarkers for BC

**Fig. 6:**
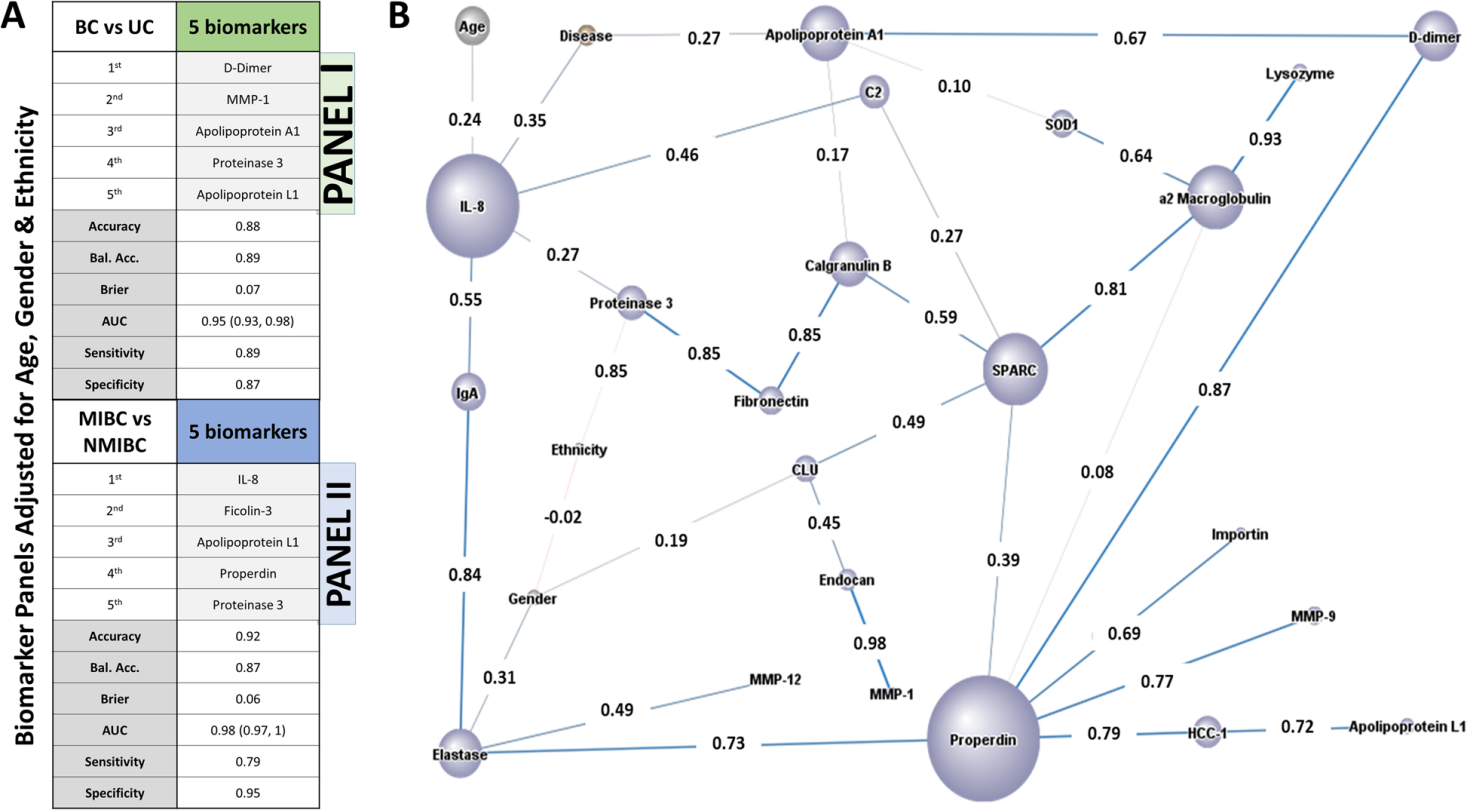
Analysis of BC biomarkers using multi-marker panels and Bayesian network analysis. **A** Panel 1 displays the top 5-biomarker panel that distinguishes BC from UC after adjusting for age, gender, and ethnicity. A positive prediction approach was implemented as detailed in the “Methods” section for panels 1 and 2. The combined statistics for the panel of markers are displayed. Panel 2 displays the top 5-biomarker panel that distinguishes MIBC from NMIBC after adjusting for age, gender, and ethnicity. **B** The 21 proteins that showed significant AUC values for BC vs UC and fold change > 1 were subjected to Bayesian network analysis using BayesiaLab. The network was constructed as detailed in the “Methods” section. The circular nodes represent the urine biomarkers (colored purple), features (colored gray), and disease (BC vs UC; colored brown). The size of each node was determined using “node force” and is proportional to its impact on the other nodes in the network, based on conditional probabilities. The arcs that interconnect the nodes were determined with Pearson’s correlation coefficient. The interconnections between nodes represent dependencies among the variables including the correlation coefficient between nodes. The thickness of the arcs is proportional to the correlation coefficient

Two multi-marker biomarker panels were constructed, after adjusting for age, gender, and ethnicity, using the positive prediction approach (Fig. 6A). Panel 1 was constructed for the BC vs UC comparison. The most discriminatory 5-marker panel consisted of urine d-dimer, MMP-1, Apolipoprotein A1, Proteinase 3, and Apolipoprotein L1, with an AUC of 0.95, a sensitivity value of 0.89, and a specificity value of 0.87. Not surprisingly, the top 3 proteins in panel 1 (d-dimer, MMP-1, Apolipoprotein A1) also ranked as the best single-marker performers, based on their individual AUC values (Fig. 4). Panel 2 was constructed for the MIBC vs NMIBC comparison. The most discriminatory 5-marker panel consisted of urine IL-8, Ficolin-3, Apolipoprotein L1, Properdin, and Proteinase 3. The panel produced an AUC of 0.98, with a sensitivity of 0.79 and a specificity of 0.95. Not surprisingly, 3 proteins in this panel (IL-8, Proteinase 3, Apolipoprotein L1) also ranked among the best single-marker performers, based on their individual AUC values (Fig. 5).

We next subjected all 21 proteins that showed significant ROC AUC values in discriminating BC from UC and clinical diagnosis to an unsupervised Bayesian network analysis. This analysis uses probability distributions to represent the inter-dependencies between all variables in a model and how they relate to one another. Figure 6B displays the derived Bayesian network, where the size of each node is representative of its impact on the other nodes in the network. Pearson’s correlation coefficient is displayed between the nodes and the interconnects represent dependencies among the variables. This network illustrates significant interactions between biomarkers, demographics, and disease status. As indicated in the Bayesian network, urine Apolipoprotein A1, d-dimer, and IL-8 had the largest direct impact on BC versus UC discrimination, consistent with the findings from the additional analytic approaches described above. In addition, SPARC (ON) and α2-macroglobulin also exercised a large impact on other nodes in this network. Thus, this independent machine learning algorithm re-affirms the diagnostic potential of Apolipoprotein A1, d-dimer, and IL-8 in BC.

### Secondary validation of urine protein biomarkers in BC urine by ELISA using a Chinese cohort

**Fig. 7:**
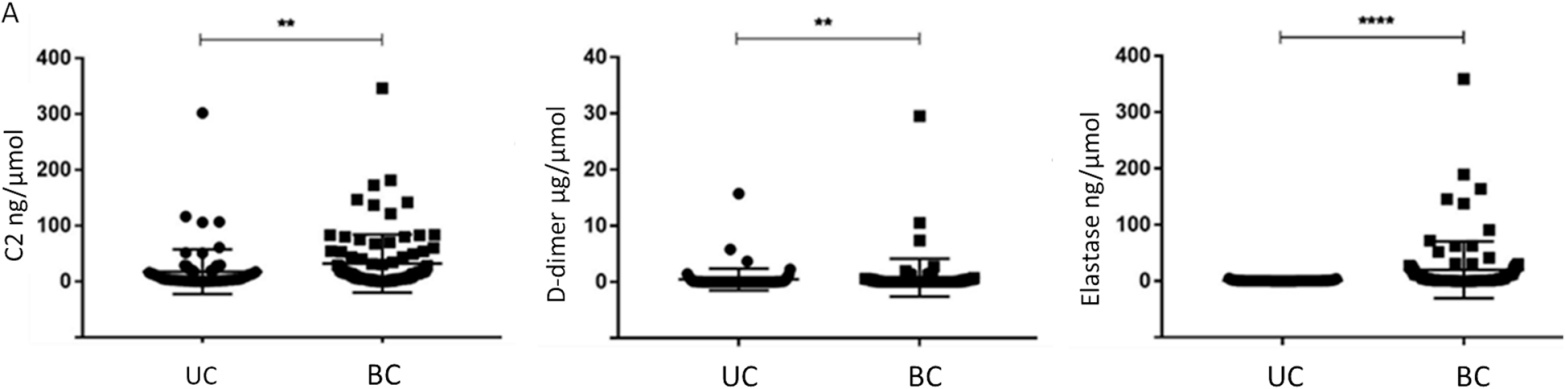
Independent ELISA validation of elevated urinary proteins in a second validation cohort of Chinese BC patients. Dot plots depict the three urine proteins that were ELISA tested in a second validation cohort of Chinese ethnicity, comprised of BC patients (*N* = 91) and UC (*N* = 77), including 19 with kidney cancer, 4 with kidney cyst, 1 with kidney harmatoma, 50 with kidney stones, 1 with fibrous epithelial polyp, and 2 with kidney angiomyolipoma, as detailed in the supplementary figures/methods. Creatinine-normalized protein values are shown for each group (black dot = UC, black square = BC). The asterisks indicate the level of significance between the groups: **p < 0.01, ****p < 0.0001

A secondary validation of upregulated urine proteins in BC vs UC was conducted in subjects of Chinese ethnicity. Three proteins were further assayed using ELISA in a cohort consisting of 77 UC and 91 BC patients (Additional file 1: Table S1). These urine proteins included complement C2, d-dimer, and Elastase which were among the best-performing markers in the first independent validation. Their associated creatinine normalized protein values are displayed in Fig. 7. The selected urine proteins were once again able to distinguish between BC and UC subjects.

## Discussion

Research over the past several years has uncovered potentially important urine biomarkers and tests for BC, including BTA and NMP22. BTA-stat, an FDA-approved urine biomarker, is used clinically to detect bladder tumor-associated antigen (human complement factor H-related protein) in the urine. A meta-analysis of BTA stat reported a specificity of 67% and a sensitivity of 75% after reviewing 13 studies [8]. The sensitivity levels of BTA-stat have been shown to positively correlate with increasing grade of BC [8]. However, BTA-stat has several limitations. These include lower specificity values and issues relating to false-positive results in benign conditions [6]. Hence, urine BTA-stat may have limitations in the diagnosis and monitoring of disease progression. Similarly, NNP22 is an FDA-approved urine biomarker designed to detect the NMP22 protein levels which are high due to cell turnover from tumor apoptosis. A meta-analysis of 19 studies has identified this marker to have a pooled specificity of 88% and a sensitivity of 56% [7].

As opposed to studies looking at an isolated protein in the urine, a few screens have been reported where multiple proteins were examined simultaneously. Summarized below are a couple of studies documenting biomarkers with both sensitivity and specificity values greater than or equal to 85%. Goodison et al. performed a validation study for the urinary concentrations of 14 proteins (A1AT, APOE, ANG, CA9, CCL18, CD44, IL-8, MMP-9, MMP-10, OPN, PAI-1, PTX3, SDC1, and VEGF) using an ELISA [30]. An 8-biomarker panel (ANG, APOE, CA-9, IL-8, MMP-9, MMP-10, PAI-1, and VEGF) achieved the most accurate BC diagnosis with a sensitivity of 92% and a specificity of 97%. However, a panel of 3 biomarkers (APOE, IL-8, and VEGF) also performed well with a sensitivity of 90% and a specificity of 97% for the detection of BC [30]. Kumar et al. identified a 5-biomarker panel consisting of Apolipoprotein A4, Coronin-1A, DJ-1/PARK7, Gamma synuclein, and Semenogelin-2. ELISA and western blot data obtained an AUC of 0.92 and 0.98, respectively, in diagnosing Ta/T1 BC (sensitivity 79.2% and 93.9% for ELISA; specificity 100% and 96.7% for western blot) [31]. For the diagnosis of T2/T3 BC, the panel of markers achieved an AUC of 0.94 and 1, respectively, using the same methods (sensitivity 86.4% and 100%; specificity 100%) [31].

Low-grade BC has a high recurrence rate; therefore, identifying biomarkers for the surveillance of BC is essential for the potential clinical management of the disease. Rosser et al. identified 10 biomarkers (ANG, APOE, CA9, IL-8, MMP-9, MMP-10, SDC1, SERPINA1, SERPINE1, and VEFGA) using ELISA for monitoring urine for recurrent BC. The complete panel achieved an AUC of 0.90, a sensitivity of 79%, and a specificity of 88% [32]. De Paoli et al. identified a panel of 6 biomarkers (cadherin-1, EN2, ErbB2, IL-6, IL-8, and VEGF-A) and three clinical parameters including BCG therapies, stage at the time of diagnosis, and past recurrences. The panel achieved an AUC of 0.91 and was identified through microarray and ELISA analysis [33].

There are several reasons to discriminate patients with bladder cancer from benign conditions. In patients with hematuria, it would be helpful to identify who needs cystoscopic evaluation which is invasive. Given that urine proteins are easily measurable and are compatible with point-of-care monitoring, a quick urine test could dramatically impact triage and workflow in urology outpatient clinics. Likewise, in bladder cancer surveillance, a reliable urine biomarker can help determine if cancer (like CIS) was missed or to avoid cystoscopy in marker-negative patients. Similarly, urine biomarkers that can reliably distinguish MIBC from NMIBC can inform us as to who has the more aggressive disease. When used as a routine point-of-care test (either at home or at outpatient visits), these urinary biomarkers may facilitate earlier identification of aggressive disease and design of tailored therapy.

The present work represents the first attempt to screen > 1000 urine proteins for urine biomarker candidates in BC, using a relatively novel aptamer-based screen. Systems biology analysis implicated molecular functions related to the extracellular matrix, collagen, integrin, heparin, and transmembrane tyrosine kinase signaling in BC susceptibility, with HNF4A and NFKB1 being key regulators. STEM analysis of the dysregulated pathways implicated a functional role for the immune system, complement, and interleukins in BC disease progression (Fig. 3D). This study has also uncovered urine proteins that outperform current FDA-approved markers in many respects. Several urine proteins (d-dimer, Apolipoprotein A1, MMP-1, Properdin, Calgranulin B) significantly discriminate BC from UC with AUC values from 0.85 to 0.96 (*p*-value < 0.0001). As a single biomarker, urine d-dimer was able to discriminate BC from UC with 96% accuracy (sensitivity = 95%; specificity = 90%). Likewise, several urine proteins (IL-8, IgA, Fibronectin, C2, Proteinase 3) significantly discriminate MIBC from NMIBC with AUC 0.84–0.99 (*p*-value < 0.001). Interestingly, several of the proteins described above have been documented to be elevated in bladder cancer tissue (at the RNA or protein level) and/or implicated in tumor biology at some level, as summarized in Additional file 1: Table S5. Considering their biomarker potential and functional properties based on the literature (Additional file 1: Table S5), these urine proteins warrant further investigation, including, d-dimer [34], Apolipoprotein A1 [35, 36], Apolipoprotein L1, Calgranulin B [37, 38], complement C2 [39], Fibronectin [40–43], Ficolin-3, IL-8 [44–49], IgA [50], MMP-1 [51, 52], Properdin, and Proteinase 3 [53]. A summary of previous research on these proteins can be found in Additional file 1: Table S6. Additional markers increased in tissues are described in Additional file 1: Table S7.d-dimer is a specific cleavage product of fibrin and a symbol of hyperfibrinolysis [34]. It is the primary diagnostic tool in various diseases, such as deep venous thrombosis, systemic illness, and cancers [54]. Previous studies have reported that molecules in the coagulation/fibrinolysis system, especially plasma fibrinogen and d-dimer, are abnormal in cancer patients [34]. In the present study, urine d-dimer levels show a significant ability to differentiate BC from UC (AUC = 0.96) (*p* < 0.0001). After correcting for patient demographics, urine d-dimer is still eligible for inclusion within the 5-biomarker panel for best distinguishing BC from UC. Perhaps most impressive is the observation that urine d-dimer demonstrates a high sensitivity for the detection of BC (95%), and at a fixed specificity of 0.8, it can achieve a sensitivity of 0.97. Hence, as a single biomarker, urine d-dimer outperforms current FDA-approved biomarkers and competing biomarkers in the research literature as a sensitive biomarker for BC detection.

Apolipoproteins (Apolipoprotein A1 and Apolipoprotein L1) are proteins known to interact with the lipids of the lipoprotein core and also the aqueous environment of the plasma. Apolipoprotein A1 is the primary protein component of high-density lipoprotein while Apolipoprotein L1 is a minor component. Previous studies have validated Apolipoprotein A1 as a novel urinary biomarker for BC [35, 36]. In the current research, Apolipoprotein A1 was the second-best performing protein in terms of the AUC value (0.91) in distinguishing BC from UC.

After adjusting for demographics, this protein ranked within a 5-marker panel for distinguishing BC from UC. Similarly, Apolipoprotein L1 also ranked within the 5-marker panel for distinguishing BC from UC and MIBC from NMIBC.

Calgranulin B (S100A9) is a zinc- and calcium-binding protein that plays a prominent role in regulating inflammatory and immune responses. Several S100 proteins, including S100A9, have received attention regarding their possible role in tumor development and progression and studies report an increased expression in a variety of tumors, including ovarian, colon, gastric, and prostate cancer [37]. Increased expression of S100A9 protein in the serum has been previously associated with tumor grade [37]. Current validation results of Calgranulin B are promising as it was among the top markers in discriminating BC from UC with an AUC of 0.85.

Complement proteins may promote tumor growth in the context of chronic inflammation [39]. Complement C2’s relation to BC at this time is unknown. However, the present study identified this protein as the fourth best single protein for differentiating MIBC from the NMIBC stage. Properdin is also a member of the complement system, controlling the alternate pathway of complement activation. Research on properdin in BC is limited. However, in the current study, properdin demonstrated the third highest AUC value (AUC = 0.89, *p* < 0.0001) in discriminating BC from UC. These biomarker findings are consistent with the observation that changes in complement activation constitute one of the major pathways that predict BC disease progression, based on STEM analysis (Fig. 3D).

Fibronectin is a glycoprotein component of the extracellular matrix. Tumor cells can attach to fibronectin via integrins or other cell surface receptors [55]. Its effectiveness as a urine biomarker for BC has been explored in a variety of studies [40–43]. Here, fibronectin showed the third best discriminatory ability in identifying MIBC compared to NMIBC. The marker exhibited an AUC value of 0.87 (*p* < 0.0001).

IL-8 is a proinflammatory CXC chemokine. It has previously been associated with the promotion of neutrophil chemotaxis and degranulation [56]. Increased expression of IL-8 has been associated with endothelial cells, infiltrating neutrophils, tumor-associated macrophages, and cancer cells [56]. Therefore, IL-8 may be a significant regulatory factor within the tumor microenvironment. Previous studies have identified urinary IL-8 as a potential marker for BC [44–49]. In the present study, urine IL-8 was the best-performing protein in the MIBC vs NMIBC comparison, with an AUC of 0.99 (*p* < 0.0001), although its specificity was modest at a fixed sensitivity of 80%. Taken together with a wealth of supporting literature, this marker has the potential to be a monitoring tool for BC disease progression and warrants further analysis in this context.

IgA is an immunoglobulin and is often the first line of defense in the resistance against infections, particularly in mucosal tissues. A correlation of intra-tumor IgA1 and poor overall survival in BC patients has been identified in a previous study [50]. However, research regarding IgA in BC urine is limited. The data presented in this study indicated that urinary IgA may differentiate MIBC from NMIBC (AUC = 0.89, *p* < 0.0001). Overall, IgA performed 2nd best out of a total of 30 urinary markers validated for this comparison. Of particular note, urine IgA exhibited the highest specificity of 80% for MIBC, at 80% sensitivity, out-performing IL-8.

Matrix metalloproteinases (MMP) are a group of zinc-dependent proteolytic enzymes. Their role involves remodeling of the extracellular matrix. Many studies have evaluated the levels of MMPs in cancer patients and have reported the vital roles of some MMPs as potential diagnostic and prognostic biomarkers in tumorigenesis [57]. The current study has uncovered MMP-1 as the fourth best-performing molecule for distinguishing BC from UC (AUC = 0.89, *p* < 0.0001). This protein was also included in a 5-marker panel for distinguishing BC from UC. At the mechanistic level, one can envision tissue matrix remodeling as an important pre-requisite for cancer progression.

## Conclusions

There is a need for these findings to be validated in additional cohorts. However, the urine proteins reported in this research exhibit great potential for use in a clinical setting. d-dimer, Apolipoprotein A1, MMP-1, Properdin, and Calgranulin B were identified as the most discriminatory urine markers in distinguishing BC from UC. Given that urine d-dimer has 97% sensitivity (at 80% specificity) for detecting BC, it may have a role in the initial diagnosis of BC, or for the detection of BC recurrence during surveillance follow-up. Urine Apolipoprotein A1, Properdin, and MMP-1 are the next best-performing biomarkers in this respect. On the other hand, urine IL-8 and IgA may have the potential in identifying disease progression during BC patient follow-up.

Several aspects of this study could be improved. Our study is limited by the relatively small sample size and the imbalance between the number of patients with NMIBC and MIBC. Because of these limitations, the generalizability of the study’s findings warrants caution. This calls for future investigation in larger patient populations. Additionally, a larger sample size could uncover markers that are less discriminatory. Given that the current study only pursued the validation of 34 urine protein biomarkers, a larger number of additional proteins found to be significant could be assessed for validation in future studies. Urine d-dimer, MMP-1, Apolipoprotein A1, Proteinase 3, and Apolipoprotein L1 need to be validated independently and in multi-marker panels in additional cross-sectional and longitudinal cohorts to confirm if they are superior to current FDA-approved markers for BC detection. Urine IL-8, Ficolin-3, Apolipoprotein L1, Properdin, and Proteinase 3 also need to be validated both independently and as a multi-marker panel in future cross-sectional and longitudinal cohorts to confirm if they are good indicators for bladder cancer disease progression. These novel urine biomarkers for BC also warrant systemic testing to assess their utility in BC surveillance and in predicting or monitoring response to treatment.

## Supplementary Information

**Additional file 1: Fig. S1.** Consort diagram outlining the flow of the study. **Fig. S2.** Principal component analyses of the SOMAscan results. **Fig. S3.** Correlation analysis of the top 93 proteins elevated in BC urine. Fig. S4. Box plot expression profiles of the 30 ELISA validated proteins (BC vs UC). **Fig. S5.** Box plot expression profiles of the 30 ELISA validated proteins (MIBC vs NMIBC). **Table S1.** Demographic and clinical information of the secondary validation cohort of Chinese ethnicity. **Table S2.** ELISA validation kits selected for 34 proteins. **Table S3.** Single marker AUC analysis for the comparison of BC vs UC. **Table S4.** Single marker AUC analysis for the comparison of MIBC vs NMIBC. **Table S5.** Literature and public database profiles of shortlisted urine proteins. **Table S6.** Literature profiles of the outstanding proteins in urine and serum. **Table S7.** Literature profiles of the outstanding proteins in tissues.

## Data Availability

Supplementary information is available for this paper. All primary data will be made available by the corresponding author, if not in the supplementary data. The proteomic data will be made available upon request from the corresponding author.

## Abbreviations

AUC: Area under the curve
BC: Bladder cancer
DAVID: Database for Annotation, Visualization, and Integrated Discovery
ELISA: Enzyme-linked immunosorbent assay
FDA: United States Food and Drug Administration
GO: Gene Ontology
MIBC: Muscle invasive bladder cancer
NMIBC: Non-muscle invasive bladder cancer
PCA: Principal component analysis
RFA: Random forest analysis
STEM: Short Time-series Expression Miner
