# Supplementary material for: Comprehensive proteomics and platform validation of urinary biomarkers for bladder cancer diagnosis and staging
Source: BMC Med. 2023 Apr 5;21:133. doi: 10.1186/s12916-023-02813-x (PMC10074794; doi:10.1186/s12916-023-02813-x)
Supplement: Supplementary file 1 — Additional file 1: Fig. S1. Consort diagram outlining the flow of the study. Fig. S2. Principal component analyses of the SOMAscan results. Fig. S3. Correlation analysis of the top 93 proteins elevated in BC urine. Fig. S4. Box plot expression profiles of the 30 ELISA validated proteins (BC vs UC). Fig. S5. Box plot expression profiles of the 30 ELISA validated proteins (MIBC vs NMIBC). Table S1. Demographic and clinical information of the secondary validation cohort of Chinese ethnicity. Table S2. ELISA validation kits selected for 34 proteins. Table S3. Single marker AUC analysis for the comparison of BC vs UC. Table S4. Single marker AUC analysis for the comparison of MIBC vs NMIBC. Table S5. Literature and public database profiles of shortlisted urine proteins. Table S6. Literature profiles of the outstanding proteins in urine and serum. Table S7. Literature profiles of the outstanding proteins in tissues. [file 12916_2023_2813_MOESM1_ESM.docx]

**Comprehensive Proteomics and Platform Validation of**

**Urinary Biomarkers for Bladder Cancer Diagnosis and Staging**

**Additional file 1: Fig. S1**. **Consort diagram outlining the flow of the study.** A consort diagram outlining the flow of the study. The initial aptamer-based screen of BC urine samples for 1317 proteins was completed and underwent data analysis to identify protein biomarkers for validation. 1317 proteins were narrowed down to 30 urine proteins for validation in the independent cohort, using ELISA. Of the 30 proteins chosen for validation in the independent cohort, 3 of the top proteins were chosen for a second validation in the Chinese cohort. A panel of 5 biomarkers was created based on the first independent validation of BC vs UC and MIBC vs NMIBC with adjustments for age, gender, and ethnicity. The 5-marker panel for BC vs UC discrimination consisted of D-dimer, MMP-1, Apolipoprotein A1, Proteinase 3, and Apolipoprotein L1. Whereas the 5-marker panel for MIBC vs NMIBC discrimination consisted of IL-8, Ficolin-3, Apolipoprotein L1, Properdin, and Proteinase 3. These urine biomarkers were identified as the best discriminatory proteins for their respective comparisons.

**Additional file 1: Fig. S2. Principal component analyses of the SOMAscan results**. A) A 2D PCA plot of all subjects, using the 1,262 expressed proteins. Proteins were considered not expressed if present in the bottom 5^th^ percentile of BC and UC subjects. B) A 2D PCA plot of all subjects, using the 330 proteins that were differentially expressed (BC vs UC, Mann Whitney p-value < 0.05). A, B) Bladder cancer is represented by a red circle while urology control is represented by a green circle. The first two principal components are displayed on each axis of the plot. C) A 2D PCA plot of all subjects, using the 1,262 expressed proteins. Proteins were considered not expressed if present in the bottom 5^th^ percentile of BC and UC subjects. D) A 2D PCA plot of all subjects, using the 330 proteins that were differentially expressed (BC vs UC, Mann Whitney p-value < 0.05). C, D) Bladder cancer is represented by a red circle while urology control is represented by a green circle. The first two principal components are displayed on each axis of the plot.

**Additional file 1: Fig. S3. Correlation analysis of the top 93 proteins elevated in BC urine.** Correlation plot displaying the expression profiles of the top 93 proteins (BC vs UC, Mann Whitney p-value < 0.05, fold change > 2) elevated in BC urine across the entire cohort. Pearson’s and Spearman’s correlation coefficient were determined for each pair. The proteins were ordered based on hierarchical clustering. Each circle represents the correlation for a protein pair. Blue corresponds to positive correlation while red corresponds to negative correlation.


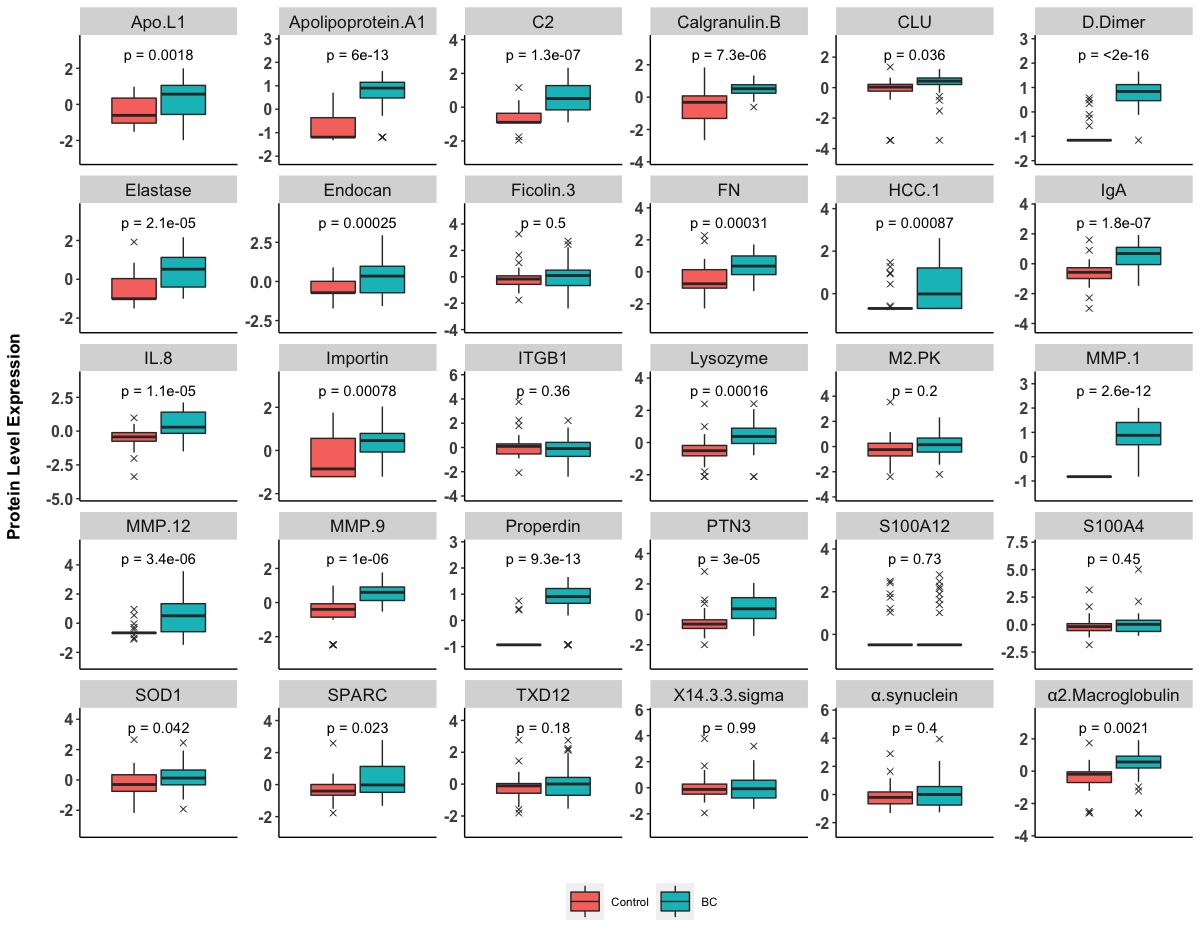


**Additional file 1: Fig. S4.** **Box plot expression profiles of the 30 ELISA validated proteins (BC vs UC).** Independent ELISA validation of urinary proteins in bladder cancer versus urology control. Box plot results of urine proteins markers in BC (N=37) and UC (N=31), as assayed by ELISA. The y-axis represents the protein level expression value after standardization. The levels of each protein were standardized to have a mean of zero and a unit standard deviation, after applying log_2_-transformation. P-values obtained from a t-test are displayed for each biomarker. UC is represented by a pink box while BC is represented by a blue box. Outliers are depicted by an X.


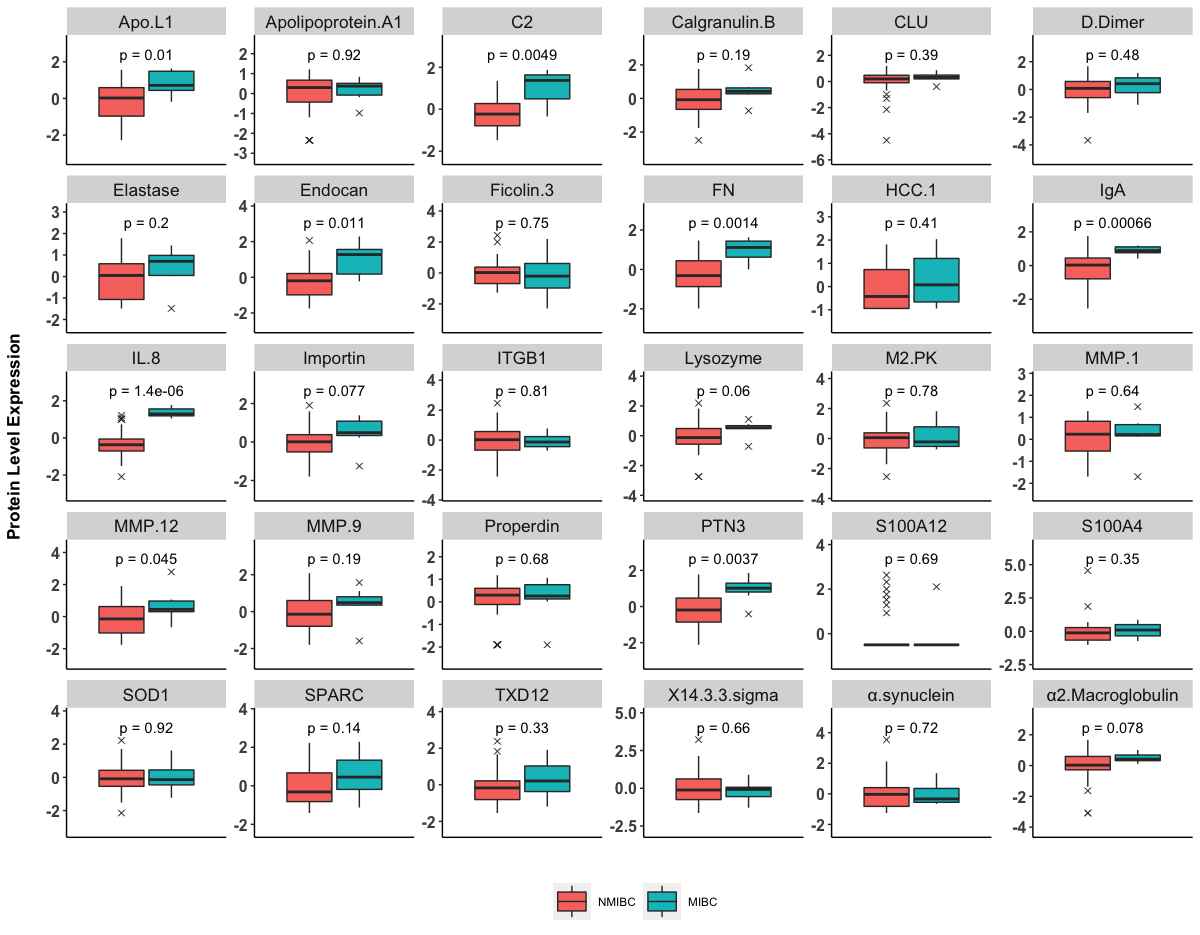


**Additional file 1: Fig. S5**. **Box plot expression profiles of the 30 ELISA validated proteins (MIBC vs NMIBC).** Independent ELISA validation of 30 urinary proteins in MIBC versus NMIBC. Box plot results of urine proteins markers in BC (N=37), segregated by muscle invasion. The numbers of patients with NMIBC and MIBC stages were 30 and 7, respectively. The y-axis represents the protein level expression value after standardization. The levels of each protein were standardized to have a mean of zero and a unit standard deviation, after applying log_2_-transformation. P-values obtained from a Wilcoxon Rank Sum Test are displayed for each biomarker. NMIBC is represented by a pink box while MIBC is represented by a blue box. Outliers are depicted by an X.

| **Additional file 1: Table S1.** Demographic and clinical information of the secondary validation cohort of Chinese ethnicity | | | | | |  |  |
| --- | --- | --- | --- | --- | --- | --- | --- |
| **Variable** | **Category** | | **UC** | **BC** | |  |  |
|  |  | | **(N=77)** | **(N=91)** | |  |  |
| Age* |  | | 55.86±13.239 | 63.52±10.524 | |  |  |
|  |  | |  |  | |  |  |
| Gender, n (%) | Male | | 48(62.3%） | 72(79.1%） | |  |  |
|  | Female | | 29(37.7%） | 19(20.9%） | |  |  |
|  |  | |  |  | |  |  |
| Ethnicity, n (%) | Asian | | 77(100%） | 91(100%） | |  |  |
|  |  | |  |  | |  |  |
| Diagnosis | Fibrous epithelial polyp | | 1(1.3%) | N/A | |  |  |
|  | Kidney angiomyolipoma | | 2(2.6%) | N/A | |  |  |
|  | Kidney cancer | | 19(24.7%) | N/A | |  |  |
|  | Kidney cyst | | 4(5.2%) | N/A | |  |  |
|  | Kidney harmatoma | | 1(1.3%) | N/A | |  |  |
|  | Kidney Stone | | 50(64.9%) | N/A | |  |  |
|  | Bladder cancer | | N/A | 84(92.3%) | |  |  |
|  | Renal pelvic carcinoma | | N/A | 1(1.1%) | |  |  |
|  | Ureteral carcinoma | | N/A | 1(1.1%) | |  |  |
|  | Uroepithelium carcinoma | | N/A | 5(5.5%) | |  |  |
|  | |  | | |  | |  |

* Age displayed as mean ± standard deviation

Demographic information pertaining to the 168 subjects whose urine samples were used for the second ELISA validation cohort of Chinese ethnicity. UC comprised of 77 subjects while BC comprised of 91 subjects. Most patients regarded as BC were diagnosed with bladder cancer except for 7 patients with urothelial cancers (including ureteric cancer).

| **Additional file 1: Table S2.** ELISA validation kits selected for 34 proteins | | | |  |  |  |
| --- | --- | --- | --- | --- | --- | --- |
| **Protein** | **ELISA Manufacturer** | **Urine Dilution** | **Reason for Selection** | **Outcome of ELISA** | |  |
|  |  |  |  |  |  |  |
| 14-3-3 sigma | RayBiotech | 1:5 | Top 93 in SOMAScan BC vs. UC | ELISA worked but not increased in BC | |  |
| 3HAO | MyBiosource | NA | Selected from correlation clusters | Didn't pass quality control | |  |
| α-synuclein | RayBiotech | 1:2 | Selected from correlation clusters | ELISA worked but not increased in BC | |  |
| α2 Macroglobulin | RayBiotech | 1:50 | Selected from correlation clusters | Validated by ELISA | |  |
| Apolipoprotein A1 | Abcam | 1:5 | Selected from correlation clusters and random forest analysis | Validated by ELISA | |  |
| Apolipoprotein L1 | RayBiotech | 1:20 | Selected from correlation clusters | Validated by ELISA | |  |
| C2 | LifeSpan Biosciences | 1:50 | Selected from correlation clusters | Validated by ELISA | |  |
| Calgranulin B | RayBiotech | 1:50 | Selected from correlation clusters and random forest analysis | Validated by ELISA | |  |
| CLU | RayBiotech | 1:100 | Implicated in public databases | ELISA worked but not increased in BC | |  |
| Cofilin-1 | RayBiotech | NA | Selected from correlation clusters | Didn't pass quality control | |  |
| Cytochrome c | RayBiotech | NA | Selected from correlation clusters | Didn't pass quality control | |  |
| D-dimer | RayBiotech | 1:100 | Top 93 in SOMAScan BC vs. UC | Validated by ELISA | |  |
| Elastase | RayBiotech | 1:5 | Selected from correlation clusters | Validated by ELISA | |  |
| Endocan | Abcam | 1:10 | Selected from correlation clusters | Validated by ELISA | |  |
| Fibronectin | RayBiotech | 1:5 | Top 93 in SOMAScan BC vs. UC | Validated by ELISA | |  |
| Ficolin-3 | RayBiotech | 1:25 | Selected from correlation clusters | ELISA worked but not increased in BC | |  |
| HCC-1 | RayBiotech | 1:10 | Implicated in public databases | Validated by ELISA | |  |
| HMG-1 | MyBiosource | NA | Selected from correlation clusters | Didn't pass quality control | |  |
| IgA | RayBiotech | 1:100 | Top 93 in SOMAScan BC vs. UC | Validated by ELISA | |  |
| IL-8 | Abcam | 1:10 | Selected from correlation clusters | Validated by ELISA | |  |
| Importin | RayBiotech | 1:2 | Implicated in public databases | Validated by ELISA | |  |
| ITGB1 | RayBiotech | 1:2 | Selected from correlation clusters | ELISA worked but not increased in BC | |  |
| Lysozyme | RayBiotech | 1:50 | Top 93 in SOMAScan BC vs. UC | Validated by ELISA | |  |
| M2PK | Abcam | 1:10 | Top 93 in SOMAScan BC vs. UC | ELISA worked but not increased in BC | |  |
| MMP-1 | RayBiotech | 1:5 | Selected from correlation clusters | Validated by ELISA | |  |
| MMP-12 | RayBiotech | 1:5 | Selected from correlation clusters | Validated by ELISA | |  |
| MMP-9 | RayBiotech | 1:100 | Top 93 in SOMAScan BC vs. UC | Validated by ELISA | |  |
| Properdin | RayBiotech | 1:5 | Selected from correlation clusters and random forest analysis | Validated by ELISA | |  |
| Proteinase 3 | RayBiotech | 1:50 | Selected from correlation clusters | Validated by ELISA | |  |
| S100A12 | RayBiotech | 1:5 | Selected from correlation clusters | ELISA worked but not increased in BC | |  |
| S100A4 | RayBiotech | 1:2 | Selected from correlation clusters | ELISA worked but not increased in BC | |  |
| SOD1 | RayBiotech | 1:50 | Selected from correlation clusters and random forest analysis | ELISA worked but not increased in BC | |  |
| SPARC | RayBiotech | 1:10 | Implicated in public databases | Validated by ELISA | |  |
| TXD12 | RayBiotech | 1:10 | Most significant p-value in SOMAScan BC vs. UC | ELISA worked but not increased in BC | |  |

Rationale for selection of proteins for ELISA validation: Seven protein were selected for validation based on being in the top 93 proteins in the BC vs UC SOMAScan. Proteins were order based upon Mann Whitney p-value and fold change. Eighteen proteins were selected for validation from correlation clusters alone while 4 proteins were selected from both correlation clusters and random forest analysis. Correlation clusters were identified for the top 50 proteins in the BC vs UC SOMAScan. The four proteins identified through random forest were among the top 10 most discriminatory proteins according to their GINI coefficient. Four proteins were implicated in public databases. One protein was selected for further validation due to having the most significant p-value in the SOMAScan BC vs UC.

**Additional file 1: Table S3.** Single marker AUC analysis for the comparison of BC vs UC

| **Protein** | **Brier Score** | **Specificity** | **Sensitivity** | **Accuracy** | **Lower Accuracy** | **Upper Accuracy** | **Balanced Accuracy** | **AUC** | **Lower AUC** | **Upper AUC** |
| --- | --- | --- | --- | --- | --- | --- | --- | --- | --- | --- |
| **D-Dimer** | **0.07** | **0.91** | **0.94** | **0.92** | **0.83** | **0.98** | **0.92** | **0.96** | **0.92** | **1** |
| **Apolipoprotein A1** | **0.12** | **0.86** | **0.83** | **0.84** | **0.73** | **0.92** | **0.85** | **0.91** | **0.83** | **0.98** |
| **MMP-1** | **0.1** | **1** | **0.78** | **0.88** | **0.78** | **0.95** | **0.89** | **0.89** | **0.82** | **0.96** |
| **Properdin** | **0.12** | **0.9** | **0.81** | **0.85** | **0.74** | **0.93** | **0.86** | **0.89** | **0.82** | **0.96** |
| **IgA** | **0.16** | **0.87** | **0.73** | **0.79** | **0.67** | **0.88** | **0.8** | **0.86** | **0.76** | **0.96** |
| **MMP-9** | **0.16** | **0.8** | **0.75** | **0.77** | **0.65** | **0.86** | **0.78** | **0.85** | **0.76** | **0.94** |
| **Calgranulin B** | **0.16** | **0.77** | **0.84** | **0.8** | **0.69** | **0.89** | **0.81** | **0.85** | **0.75** | **0.95** |
| **C2** | **0.17** | **0.8** | **0.72** | **0.75** | **0.63** | **0.85** | **0.76** | **0.84** | **0.74** | **0.93** |
| **Proteinase 3** | **0.19** | **0.84** | **0.7** | **0.76** | **0.64** | **0.85** | **0.77** | **0.81** | **0.71** | **0.92** |
| **Lysozyme** | **0.2** | **0.77** | **0.73** | **0.74** | **0.62** | **0.84** | **0.75** | **0.81** | **0.7** | **0.92** |
| **MMP-12** | **0.18** | **0.9** | **0.61** | **0.74** | **0.62** | **0.84** | **0.76** | **0.8** | **0.69** | **0.91** |
| **IL-8** | **0.19** | **0.78** | **0.72** | **0.74** | **0.61** | **0.84** | **0.75** | **0.8** | **0.7** | **0.91** |
| **Elastase** | **0.2** | **0.67** | **0.73** | **0.7** | **0.57** | **0.8** | **0.7** | **0.79** | **0.68** | **0.9** |
| **α2 Macroglobulin** | **0.22** | **0.78** | **0.81** | **0.78** | **0.66** | **0.87** | **0.79** | **0.78** | **0.66** | **0.9** |
| **Fibronectin** | **0.21** | **0.68** | **0.7** | **0.68** | **0.55** | **0.79** | **0.69** | **0.77** | **0.65** | **0.89** |
| **CLU** | **0.24** | **0.51** | **0.78** | **0.63** | **0.5** | **0.74** | **0.64** | **0.74** | **0.62** | **0.86** |
| **Endocan** | **0.22** | **0.7** | **0.7** | **0.69** | **0.56** | **0.8** | **0.7** | **0.74** | **0.62** | **0.86** |
| **HCC-1** | **0.22** | **0.85** | **0.53** | **0.67** | **0.55** | **0.78** | **0.69** | **0.72** | **0.61** | **0.83** |
| **Apolipoprotein L1** | **0.23** | **0.67** | **0.64** | **0.64** | **0.52** | **0.76** | **0.65** | **0.71** | **0.59** | **0.84** |
| **Importin** | **0.22** | **0.67** | **0.7** | **0.68** | **0.55** | **0.79** | **0.69** | **0.71** | **0.58** | **0.84** |
| **SOD1** | **0.25** | **0.67** | **0.54** | **0.57** | **0.44** | **0.69** | **0.6** | **0.64** | **0.51** | **0.77** |
| **SPARC** | **0.24** | **0.67** | **0.53** | **0.57** | **0.45** | **0.69** | **0.6** | **0.63** | **0.49** | **0.76** |
| **M2-PK** | **0.26** | **0.59** | **0.56** | **0.54** | **0.41** | **0.66** | **0.57** | **0.58** | **0.45** | **0.72** |
| **TXD12** | **0.25** | **0.6** | **0.52** | **0.53** | **0.4** | **0.65** | **0.56** | **0.55** | **0.41** | **0.69** |
| **Ficolin-3** | **0.26** | **0.56** | **0.49** | **0.48** | **0.35** | **0.61** | **0.52** | **0.52** | **0.38** | **0.66** |
| **S100A4** | **0.26** | **0.59** | **0.49** | **0.5** | **0.37** | **0.62** | **0.54** | **0.51** | **0.37** | **0.65** |
| **α-synuclein** | **0.26** | **0.59** | **0.48** | **0.5** | **0.37** | **0.62** | **0.54** | **0.51** | **0.37** | **0.65** |
| **ITGB1** | **0.26** | **0.53** | **0.54** | **0.5** | **0.37** | **0.62** | **0.54** | **0.5** | **0.36** | **0.64** |
| **S100A12** | **0.26** | **0.78** | **0.19** | **0.42** | **0.3** | **0.55** | **0.49** | **0.48** | **0.38** | **0.58** |
| **14-3-3 sigma** | **0.26** | **0.36** | **0.54** | **0.42** | **0.3** | **0.55** | **0.45** | **0.44** | **0.3** | **0.58** |

Performance metrics from a bootstrap logistic regression model using only a single biomarker based on the comparison between BC and UC.

**Additional file 1: Table S4**. Single marker AUC analysis for the comparison of MIBC vs NMIBC

| **Protein** | **Brier Score** | **Specificity** | **Sensitivity** | **Accuracy** | **Lower Accuracy** | **Upper Accuracy** | **Balanced Accuracy** | **AUC** | **Lower AUC** | **Upper AUC** |
| --- | --- | --- | --- | --- | --- | --- | --- | --- | --- | --- |
| **IL-8** | **0.06** | **0.87** | **1** | **0.89** | **0.75** | **0.97** | **0.91** | **0.98** | **0.95** | **1** |
| **IgA** | **0.13** | **0.77** | **0.71** | **0.78** | **0.62** | **0.9** | **0.8** | **0.88** | **0.77** | **1** |
| **Fibronectin** | **0.12** | **0.78** | **0.88** | **0.78** | **0.62** | **0.9** | **0.8** | **0.87** | **0.75** | **0.99** |
| **C2** | **0.1** | **0.76** | **0.71** | **0.76** | **0.59** | **0.88** | **0.75** | **0.85** | **0.65** | **1** |
| **Proteinase 3** | **0.14** | **0.72** | **0.88** | **0.73** | **0.56** | **0.86** | **0.78** | **0.84** | **0.67** | **1** |
| **Endocan** | **0.13** | **0.79** | **0.48** | **0.73** | **0.56** | **0.86** | **0.68** | **0.82** | **0.66** | **0.98** |
| **Apolipoprotein L1** | **0.13** | **0.67** | **0.77** | **0.68** | **0.5** | **0.82** | **0.74** | **0.81** | **0.64** | **0.98** |
| **MMP-12** | **0.14** | **0.53** | **0.71** | **0.57** | **0.39** | **0.73** | **0.64** | **0.75** | **0.56** | **0.93** |
| **Lysozyme** | **0.16** | **0.66** | **0.88** | **0.7** | **0.53** | **0.84** | **0.75** | **0.73** | **0.49** | **0.96** |
| **Importin** | **0.16** | **0.6** | **0.79** | **0.65** | **0.47** | **0.8** | **0.74** | **0.72** | **0.48** | **0.95** |
| **α2 Macroglobulin** | **0.16** | **0.61** | **0.86** | **0.62** | **0.45** | **0.78** | **0.7** | **0.72** | **0.56** | **0.88** |
| **SPARC** | **0.15** | **0.7** | **0.62** | **0.68** | **0.5** | **0.82** | **0.69** | **0.68** | **0.42** | **0.94** |
| **Calgranulin B** | **0.16** | **0.53** | **0.71** | **0.64** | **0.46** | **0.79** | **0.72** | **0.67** | **0.44** | **0.88** |
| **MMP-9** | **0.16** | **0.61** | **0.91** | **0.65** | **0.47** | **0.8** | **0.72** | **0.64** | **0.38** | **0.89** |
| **Elastase** | **0.17** | **0.53** | **0.43** | **0.51** | **0.34** | **0.68** | **0.55** | **0.64** | **0.39** | **0.88** |
| **CLU** | **0.16** | **0.43** | **0.68** | **0.46** | **0.29** | **0.63** | **0.57** | **0.61** | **0.41** | **0.83** |
| **S100A4** | **0.16** | **0.55** | **0.48** | **0.51** | **0.34** | **0.68** | **0.59** | **0.61** | **0.38** | **0.85** |
| **TXD12** | **0.16** | **0.64** | **0.39** | **0.62** | **0.45** | **0.78** | **0.59** | **0.61** | **0.34** | **0.87** |
| **HCC-1** | **0.16** | **0.57** | **0.23** | **0.49** | **0.32** | **0.66** | **0.49** | **0.58** | **0.33** | **0.84** |
| **D-Dimer** | **0.17** | **0.5** | **0.62** | **0.53** | **0.35** | **0.69** | **0.62** | **0.56** | **0.31** | **0.81** |
| **MMP-1** | **0.16** | **0.43** | **0.62** | **0.46** | **0.29** | **0.63** | **0.62** | **0.53** | **0.3** | **0.77** |
| **14-3-3 sigma** | **0.16** | **0.47** | **0.29** | **0.41** | **0.24** | **0.58** | **0.43** | **0.52** | **0.31** | **0.75** |
| **S100A12** | **0.16** | **0.14** | **0.84** | **0.24** | **0.11** | **0.42** | **0.51** | **0.51** | **0.34** | **0.7** |
| **Properdin** | **0.16** | **0.29** | **0.45** | **0.34** | **0.19** | **0.52** | **0.49** | **0.51** | **0.25** | **0.78** |
| **ITGB1** | **0.16** | **0.53** | **0.43** | **0.51** | **0.34** | **0.68** | **0.55** | **0.5** | **0.31** | **0.69** |
| **Ficolin-3** | **0.17** | **0.48** | **0.43** | **0.49** | **0.32** | **0.66** | **0.5** | **0.49** | **0.16** | **0.78** |
| **M2-PK** | **0.16** | **0.5** | **0.29** | **0.43** | **0.27** | **0.61** | **0.45** | **0.49** | **0.22** | **0.75** |
| **Apolipoprotein A1** | **0.17** | **0.29** | **0.36** | **0.31** | **0.16** | **0.49** | **0.45** | **0.45** | **0.23** | **0.67** |
| **SOD1** | **0.16** | **0.57** | **0** | **0.49** | **0.32** | **0.66** | **0.44** | **0.38** | **0.13** | **0.63** |
| **α-synuclein** | **0.16** | **0.45** | **0.43** | **0.46** | **0.29** | **0.63** | **0.54** | **0.33** | **0.11** | **0.55** |

Performance metrics from a bootstrap logistic regression model using only a single biomarker based on the comparison between MIBC and NMIBC.

| **Additional file 1: Table S5.** Literature and public database profiles of shortlisted urine proteins | | | | | | |  |  |  |  |
| --- | --- | --- | --- | --- | --- | --- | --- | --- | --- | --- |
|  | **Public Database Search** | | | | | **Literature Search (ref)** | | | |  |
|  |  |  |  |  |  |  |  |  |  |  |
|  |  |  |  |  |  |  |  |  |  |  |
| **Protein** | **Firebrowser** | **Oncomine** | **FPKM** | **U Exosomes** | **OncoDB** | **Up in BC Serum** | **Up in BC Urine** | **Up in BC cells** | **Potential Role in BC biology** |  |
|  |  |  |  |  |  |  |  |  |  |  |
| α2 Macroglobulin | 3.4 | 1 | 43.4 |  | 0.04 | 84-85 |  |  |  |  |
| Apolipoprotein A1 | 4.2 | 1.1 | 0.1 | √ | 1 |  | 35-36, 60-63 |  | 65 |  |
| Apolipoprotein L1 | 3.7 | 12.4 | 84.8 |  | 2.30E-04 |  |  |  |  |  |
| C2 | 6.3 | 2.2 | 3.3 |  | 0.09 |  |  |  | 39 |  |
| Calgranulin B | 393 | 5.2 | 4.16E+02 | √ | 0.09 | 37-38 |  | 67 | 66 |  |
| CLU | 4.7 | 1.2 | 29.6 |  | 0.45 | 88 | 35, 86-87 | 88 | 89 |  |
| D-dimer | 2.8 | 1.5 | 8 |  | 0.1 | 34, 58 |  |  | 54, 59 |  |
| Elastase | 1.9 | 1 | 0 | √ | 0.1 |  |  |  | 90 |  |
| Endocan | 61.5 | 3.7 | 2.8 |  | 0.41 | 91-92 | 93 |  | 93 |  |
| Fibronectin | 54.2 | 2.3 | 59.7 | √ | 0.01 | 68-69 | 40-43, 69-70 |  | 55 |  |
| HCC-1 | 5.1 | 1.1 | 0.1 |  | 0.1 |  |  |  |  |  |
| IgA | N/A | 4.8 | 0 | √ | 0.6 |  | 73 | 50 |  |  |
| IL-8 | 68.4 | 3.6 | 6.6 |  | 0.31 |  | 44-46, 48-49, 72 | 47 | 56, 71 |  |
| Importin | 2 | 2.8 | 35.3 |  | 0.25 |  |  | 97-98 | 94-96 |  |
| Lysozyme | 8.8 | 1.1 | 19.1 | √ | 0.4 |  |  |  | 99 |  |
| MMP-1 | 102 | 7.6 | 9.5 |  | 0.78 | 76 | 51-52 | 75 | 57, 74 |  |
| MMP-12 | 67.1 | 5.1 | 3.7 |  | 0.32 | 102 |  | 75 | 57, 74 |  |
| MMP-9 | 101 | 7.6 | 9.5 |  | 0.1 | 102, 76-77 | 38, 81-82 | 47, 75, 79-80 | 57, 74, 78 |  |
| Properdin | 3.6 | 1 | 0.2 |  | 0.75 |  |  |  | 83 |  |
| Proteinase 3 | 4.6 | 1.3 | 0 | √ | 0.71 |  | 53 |  |  |  |
| SOD1 | 1.5 | 2.1 | 100.2 | √ | 0.02 | 100 |  | 101 |  |  |

Firebrowser: RNA level fold change ratio of BC to control

Oncomine: RNA level fold change; listed is the highest value identified.

FPKM: Median FPKM (Fragments per kilobase of exon model per million reads mapped) for gene expression.

U Exosomes: Present in ExoCarta Urine database.

OncoDB: BC Log Rank Test p-value, indicative of a significant impact on survival

**Additional file 1: Table S6. Literature profiles of the outstanding proteins in urine and serum.**

| **Protein** | **Specimen** | **Subjects** | **Results** | **Source** |  |
| --- | --- | --- | --- | --- | --- |
| **Apolipoprotein A1** | Urine | 32 healthy and benign subjects. 40 subjects with BC. | Increased expression in patients with BC. AUC = 0.928 p < 0.01. | 35 |  |
|  | Urine | 223 subjects with BC. 156 subjects without BC. | Sensitivity = 89.2%; Specificity = 84.6% in diagnosing BC. | 36 |  |
|  | Urine | 50 healthy subjects. 50 with cystitis. 50 subjects with BC. | Significantly increased in the malignant group compared to benign and control subjects. AUC = 1 | 60 |  |
|  | Serum |  | AUC = 0.629. |  |  |
|  | Urine | 126 subjects total. 76 subjects with BC. | AUC = 0.982; p<0.001 for diagnosis of BC. | 61 |  |
|  | Urine | Four groups of subjects. Control group, benign bladder disease group, low-grade malignant BC group, and high-grade malignant BC group. | Significantly higher in BC groups than control groups (p < 0.01). Sensitivity of 91.6% and specificity of 85.7% to diagnose BC. | 62 |  |
|  |  |  |  |  |  |
|  | Urine | 12 subjects with urothelial neoplasm of the urinary bladder. 3 control samples and 4 normal samples. | FC = 2.6 in high-grade BC. | 63 |  |
|  |  |  |  |  |  |
| **Calgranulin B** | Serum | 2 subjects with BC. | Increased expression of S100A9 protein was associated with tumor grade (p < 0.05). | 37 |  |
|  | Serum | 33 subjects with low-grade BC and 32 with high-grade BC. 25 healthy controls. | S100A8 and S100A9 combined produced an AUC = 0.946 to distinguish BC from healthy controls. | 38 |  |
| **D-dimer** | Serum | 206 subjects with NMIBC. | High D-dimer levels were significantly related to poor recurrence free survival (p < 0.001) and progression free survival (p < 0.001). D-dimer levels were increased with advanced pathologic T stage (p = 0.026), large tumor size (p = 0.012), and multiple tumor lesions (p = 0.006). | 34 |  |
|  |  |  |  |  |  |
|  |  |  |  |  |  |
|  |  |  |  |  |  |
|  | Serum | 232 subjects with upper tract urothelial carcinoma. | Plasma D-dimer was significantly associated with advanced tumor status (size, location, hydronephrosis, tumor grade, lymph node involvement, grade, and stage) p < 0.05. | 58 |  |
|  |  |  |  |  |  |
| **Fibronectin** | Urine | 68 subjects with BC. 10 subjects with benign urologic disease and 45 healthy subjects. | Subjects with BC had significantly greater levels than healthy subjects (p < 0.001). | 41 |  |
|  |  |  |  |  |  |
|  | Urine | 931 urine samples belonging to 402 subjects. 112 subjects under suspicion for a primary bladder tumor. 104 BC patients receiving intravesical instillations. 45 subjects with other urological diseases. 32 healthy subjects. | Overall sensitivity of 80% at specificities of 88.4 and 74.7%. | 42 |  |
|  |  |  |  |  |  |
|  |  |  |  |  |  |
|  | Urine | 100 subjects with BC. 93 subjects with benign urological conditions. 47 healthy subjects. | Sensitivity = 82; specificity = 84.3; PPV = 78.8; NPV = 86.8; Accuracy = 83.3. | 43 |  |
|  |  |  |  |  |  |
|  | Urine | 35 subjects with BC and 35 healthy subjects. | Significantly increased in BC. AUC = 0.976. | 69 |  |
|  | Serum |  | Significantly increased in BC. AUC = 0.966. |  |  |
|  | Urine | 106 subjects with BC. 13 subjects with benign urological conditions. 24 healthy control subjects. | Significantly increased in BC compared to benign and healthy subjects. | 70 |  |
|  |  |  |  |  |  |
|  | Serum | 20 subjects with transitional cell carcinoma of the bladder and 20 control subjects with benign urological conditions. | Subjects with TCC of the bladder showed significantly higher levels than the control group (p < 0.05). | 68 |  |
|  |  |  |  |  |  |
| **IL-8** | Urine | 64 BC subjects and 63 non-cancer subjects. | Significantly elevated in BC subjects (AUC = 0.79; 95% confidence interval 0.72-0.86). | 44 |  |
|  | Urine | 130 subjects with intermediate and high-risk NMIBC | Nine cytokines (IL-2, IL-8, IL-6, IL-1ra, IL-10, IL-12[p70], IL-12[p40], TRAIL, and TNF-α) predicted the likelihood of recurrence with 85.5% accuracy. | 45 |  |
|  |  |  |  |  |  |
|  | Urine | 72 subjects with urothelial cancer and 42 healthy controls. | Significantly elevated in patients compared to healthy controls (p = 0.002). | 46 |  |
|  | Urine | 28 subjects with superficial BC. 24 subjects with MIBC. 24 healthy control subjects. | IL-8 was significantly increased in MIBC subjects when compared to superficial BC subjects and healthy subjects (p < 0.001). AUC ranging from 0.739 to 0.967 for discriminating BC from healthy subjects. | 48 |  |
|  |  |  |  |  |  |
|  |  |  |  |  |  |
|  | Urine | 51 subjects with transitional cell carcinoma (BC). 17 subjects with prostate cancer. 23 subjects with successfully treated transitional cell carcinoma. 49 healthy subjects. | Significantly increased in BC compared to controls (p < 0.05). | 49 |  |
|  |  |  |  |  |  |
|  | Urine | 18 healthy control subjects. 20 subjects with hematuria. 50 subjects with NMIBC. 18 subjects with MIBC. | Significantly increased in BC compared to controls (p < 0.05). | 72 |  |
|  |  |  |  |  |  |
| **IgA** | Urine | 24 subjects with BC. 22 subjects with symptoms of urinary tract infection. 35 subjects without BC or symptoms of urinary tract infection. | Significantly increased in BC compared to controls (p < 0.05). | 73 |  |
|  |  |  |  |  |  |
| **MMP-1** | Serum | 29 BC subjects and 30 healthy individuals. | MMP-1 was significantly higher by 1.21-, 1.37-, 1.67- and 2.07-fold for Stage I, II, III and IV BC subjects, respectively. The levels of MMP-1 were correlated with increasing cancer stage. | 76 |  |
|  |  |  |  |  |  |
|  | Urine | 32 BC subjects and 17 patients with no detectable tumor at cystoscopy. | Significantly increased compared to subjects with no tumors (p = 0.003). | 51 |  |
|  | Urine | 131 subjects with BC. 5 subjects with prostate cancer. 33 subjects with benign lower urinary tract disorders. 36 healthy control subjects. | Significantly elevated in patients with T2-T4 BC compared to cis/Ta/T1 BC (p = 0.04 and 0.0074, respectively). | 52 |  |
|  |  |  |  |  |  |
| **Proteinase 3** | Urine | 23 subjects with benign urogenital disease. 74 subjects with BC (25T2+, 23 T1, 26Ta). | Overexpressed in subjects with T2+BC compared to T1, Ta, and benign diseases (p < 0.05). | 53 |  |

**Additional file 1: Table S7. Literature profiles of the outstanding proteins in tissues.**

| **Protein** | **Subjects** | **Results** | **Source** |  |
| --- | --- | --- | --- | --- |
| Calgranulin B | 18 benign and 32 BC tissues. | Expression elevated in tumor tissues 2-fold (p < 0.05). | 67 |  |
| CLU | 46 subjects with NMIBC. | The association between CLU and clinical factors was observed. Significant correlation with progression and muscle-invasive disease was observed (p = 0.001, p = 0.014). | 88 |  |
|  |  |  |  |  |
| IgA | 99 subjects with BC. | Correlation between intra-tumor IgA1 and poor overall survival (p = 0.011). | 50 |  |
| IL-8 | 40 BC subjects and 5 control subjects (normal tissue). | High-grade tumors exhibited significantly higher levels (p = 0.003). Invasive tumors expressed significantly higher levels (p = 0.015, p = 0.048). Tumor recurrence was significantly associated with higher levels (p = 0.005). | 47 |  |
|  |  |  |  |  |
|  |  |  |  |  |
| Importin | 234 primary Ta/T1 BC tumors treated with transurethral resection of the bladder. 377 tumors undergoing radical cystectomy. | High expression was significantly correlated with a higher risk of progression (p = 0.0002). With those undergoing radial cystectomy, high expression was correlated with a higher risk of visceral metastasis (p = 0.04). | 97 |  |
|  |  |  |  |  |
|  | 195 BC tissues. 39 clinical samples paired with normal tissue. | High expression was significantly associated with poorer prognosis (p = 0.0195). | 98 |  |
| MMP-1 | 113 urothelial carcinoma and 19 normal controls. | Significantly correlated to increasing tumor grade (p < 0.001). | 75 |  |
| MMP-12 | 113 urothelial carcinoma and 19 normal controls. | Significantly correlated to increasing tumor grade (p < 0.001). |  |  |
| MMP-9 | 40 BC subjects and 5 control subjects (normal tissue). | High-grade tumors exhibited significantly higher levels (p = 0.012). Invasive tumors expressed significantly higher than superficial tumors (p = 0.026). Tumor recurrence was significantly associated with higher levels (p = 0.003). | 47 |  |
|  |  |  |  |  |
|  |  |  |  |  |
|  | 113 urothelial carcinoma and 19 normal controls tissue. | MMP-9 was found highly expressed in tumor tissue, but just failed to show a significant correlation to tumor grade. | 75 |  |
|  |  |  |  |  |
|  | 92 BC subjects and 63 control subjects (normal tissue). | Significantly greater in BC compared to controls (p < 0.001) and significantly different between grade (p < 0.05). | 79 |  |
|  |  |  |  |  |
|  | 30 subjects with BC. | Correlated with tumor stage and associated with the invasiveness of transitional cell carcinoma (p = 0.012 and 0.023). | 80 |  |
| SOD1 | 47 transitorial cell BC samples. | Upregulated in MIBC tumors only. | 101 |  |

**References in main manuscript and/or this “Additional file”**

1. Cancer facts and statistics: American Cancer Society; [Available from: <https://www.cancer.org/research/cancer-facts-statistics/>.

2. Richters A, Aben KKH, Kiemeney LALM. The global burden of urinary bladder cancer: an update. World J Urol. 2020;38(8):1895-904.

3. Reid MD, Osunkoya AO, Siddiqui MT, Looney SW. Accuracy of grading of urothelial carcinoma on urine cytology: an analysis of interobserver and intraobserver agreement. Int J Clin Exp Pathol. 2012;5(9):882-91.

4. Sugeeta SS, Sharma A, Ng K, Nayak A, Vasdev N. Biomarkers in Bladder Cancer Surveillance. Front Surg. 2021;8:735868.

5. Wang Z, Que H, Suo C, Han Z, Tao J, Huang Z, et al. Evaluation of the NMP22 BladderChek test for detecting bladder cancer: a systematic review and meta-analysis. Oncotarget. 2017;8(59):100648-56.

6. Guo A, Wang X, Gao L, Shi J, Sun C, Wan Z. Bladder tumour antigen (BTA stat) test compared to the urine cytology in the diagnosis of bladder cancer: A meta-analysis. Can Urol Assoc J. 2014;8(5-6):E347-52.

7. Barocas DA, Boorjian SA, Alvarez RD, Downs TM, Gross CP, Hamilton BD, et al. Microhematuria: AUA/SUFU Guideline. J Urol. 2020;204(4):778-86.

8. Chang SS, Boorjian SA, Chou R, Clark PE, Daneshmand S, Konety BR, et al. Diagnosis and Treatment of Non-Muscle Invasive Bladder Cancer: AUA/SUO Guideline. J Urol. 2016;196(4):1021-9.

9. Lei R, Huo R, Mohan C. Current and emerging trends in point-of-care urinalysis tests. Expert Rev Mol Diagn. 2020;20(1):69-84.

10. Albaba D, Soomro S, Mohan C. Aptamer-Based Screens of Human Body Fluids for Biomarkers. Microarrays (Basel). 2015;4(3):424-31.

11. Sattlecker M, Kiddle SJ, Newhouse S, Proitsi P, Nelson S, Williams S, et al. Alzheimer's disease biomarker discovery using SOMAscan multiplexed protein technology. Alzheimers Dement. 2014;10(6):724-34.

12. Kiddle SJ, Sattlecker M, Proitsi P, Simmons A, Westman E, Bazenet C, et al. Candidate blood proteome markers of Alzheimer's disease onset and progression: a systematic review and replication study. J Alzheimers Dis. 2014;38(3):515-31.

13. De Groote MA, Nahid P, Jarlsberg L, Johnson JL, Weiner M, Muzanyi G, et al. Elucidating novel serum biomarkers associated with pulmonary tuberculosis treatment. PLoS One. 2013;8(4):e61002.

14. Nahid P, Bliven-Sizemore E, Jarlsberg LG, De Groote MA, Johnson JL, Muzanyi G, et al. Aptamer-based proteomic signature of intensive phase treatment response in pulmonary tuberculosis. Tuberculosis (Edinb). 2014;94(3):187-96.

15. Hathout Y, Brody E, Clemens PR, Cripe L, DeLisle RK, Furlong P, et al. Large-scale serum protein biomarker discovery in Duchenne muscular dystrophy. Proc Natl Acad Sci U S A. 2015;112(23):7153-8.

16. Ostroff RM, Bigbee WL, Franklin W, Gold L, Mehan M, Miller YE, et al. Unlocking biomarker discovery: large scale application of aptamer proteomic technology for early detection of lung cancer. PLoS One. 2010;5(12):e15003.

17. Ostroff RM, Mehan MR, Stewart A, Ayers D, Brody EN, Williams SA, et al. Early detection of malignant pleural mesothelioma in asbestos-exposed individuals with a noninvasive proteomics-based surveillance tool. PLoS One. 2012;7(10):e46091.

18. Mehan MR, Williams SA, Siegfried JM, Bigbee WL, Weissfeld JL, Wilson DO, et al. Validation of a blood protein signature for non-small cell lung cancer. Clin Proteomics. 2014;11(1):32.

19. Ganz P, Heidecker B, Hveem K, Jonasson C, Kato S, Segal MR, et al. Development and Validation of a Protein-Based Risk Score for Cardiovascular Outcomes Among Patients With Stable Coronary Heart Disease. JAMA. 2016;315(23):2532-41.

20. Stanley S, Vanarsa K, Soliman S, Habazi D, Pedroza C, Gidley G, et al. Comprehensive aptamer-based screening identifies a spectrum of urinary biomarkers of lupus nephritis across ethnicities. Nat Commun. 2020;11(1):2197.

21. Soomro S, Venkateswaran S, Vanarsa K, Kharboutli M, Nidhi M, Susarla R, et al. Predicting disease course in ulcerative colitis using stool proteins identified through an aptamer-based screen. Nat Commun. 2021;12(1):3989.

22. SomaLogic. SOMAscan Proteomic Assay Technical White Paper; 2015. p. 1–14.

23. Goksuluk Di, Korkmaz Se, Zararsiz Go, Karaağaoğlu AEr. easyROC: An Interactive Web-tool for ROC Curve Analysis Using R Language Environment. The R Journal2016. p. 213-30.

24. Catalina A, Bürkner P-C, Vehtari A. Projection Predictive Inference for Generalized Linear and Additive Multilevel Models. arXiv preprint arXiv:201006994. 2020.

25. Piironen J, Paasiniemi M, Vehtari A. Projective inference in high-dimensional problems: Prediction and feature selection. <https://doiorg/101214/20-EJS1711>. 2020;14(1):2155-97.

26. Piironen J, Paasiniemi M, Catalina A, Vehtari A. projpred: Projection Predictive Feature Selection. R package version 2.0.22020.

27. Carvalho CM, Polson NG, Scott JG, editors. Handling Sparsity via the Horseshoe. 12; 2022: PMLR.

28. Piironen J, Vehtari A. Sparsity information and regularization in the horseshoe and other shrinkage priors. <https://doiorg/101214/17-EJS1337SI>. 2017;11(2):5018-51.

29. Vehtari A, Gelman A, Gabry J. Practical Bayesian model evaluation using leave-one-out cross-validation and WAIC. Statistics and Computing. 2015;27:1413-32.

30. Goodison S, Chang M, Dai Y, Urquidi V, Rosser CJ. A multi-analyte assay for the non-invasive detection of bladder cancer. PLoS One. 2012;7(10):e47469.

31. Kumar P, Nandi S, Tan TZ, Ler SG, Chia KS, Lim WY, et al. Highly sensitive and specific novel biomarkers for the diagnosis of transitional bladder carcinoma. Oncotarget. 2015;6(15):13539-49.

32. Rosser CJ, Chang M, Dai Y, Ross S, Mengual L, Alcaraz A, et al. Urinary protein biomarker panel for the detection of recurrent bladder cancer. Cancer Epidemiol Biomarkers Prev. 2014;23(7):1340-5.

33. De Paoli M, Gogalic S, Sauer U, Preininger C, Pandha H, Simpson G, et al. Multiplatform Biomarker Discovery for Bladder Cancer Recurrence Diagnosis. Dis Markers. 2016;2016:4591910.

34. Li X, Shu K, Zhou J, Yu Q, Cui S, Liu J, et al. Preoperative Plasma Fibrinogen and D-dimer as Prognostic Biomarkers for Non-Muscle-Invasive Bladder Cancer. Clin Genitourin Cancer. 2020;18(1):11-9.e1.

35. Li H, Li C, Wu H, Zhang T, Wang J, Wang S, et al. Identification of Apo-A1 as a biomarker for early diagnosis of bladder transitional cell carcinoma. Proteome Sci. 2011;9(1):21.

36. Li C, Li H, Zhang T, Li J, Liu L, Chang J. Discovery of Apo-A1 as a potential bladder cancer biomarker by urine proteomics and analysis. Biochem Biophys Res Commun. 2014;446(4):1047-52.

37. Minami S, Sato Y, Matsumoto T, Kageyama T, Kawashima Y, Yoshio K, et al. Proteomic study of sera from patients with bladder cancer: usefulness of S100A8 and S100A9 proteins. Cancer Genomics Proteomics. 2010;7(4):181-9.

38. Bansal N, Gupta A, Sankhwar SN, Mahdi AA. Low- and high-grade bladder cancer appraisal via serum-based proteomics approach. Clin Chim Acta. 2014; 436:97-103.

39. Pio R, Corrales L, Lambris JD. The role of complement in tumor growth. Adv Exp Med Biol. 2014;772:229-62.

40. Shen Z, Wei K, Yang S, Shi S, Chen Z, Li Y, et al. Measurement of urine fibronectin in the diagnosis of invasive bladder transitional carcinoma. *Chin J Urol*. 1993;14:27-9.

41. Menéndez V, Fernández-Suárez A, Galán JA, Pérez M, García-López F. Diagnosis of bladder cancer by analysis of urinary fibronectin. Urology. 2005;65(2):284-9.

42. Sánchez-Carbayo M, Urrutia M, González de Buitrago JM, Navajo JA. Evaluation of two new urinary tumor markers: bladder tumor fibronectin and cytokeratin 18 for the diagnosis of bladder cancer. Clin Cancer Res. 2000;6(9):3585-94.

43. Eissa S, Zohny SF, Zekri AR, El-Zayat TM, Maher AM. Diagnostic value of fibronectin and mutant p53 in the urine of patients with bladder cancer: impact on clinicopathological features and disease recurrence. Med Oncol. 2010;27(4):1286-94.

44. Urquidi V, Chang M, Dai Y, Kim J, Wolfson ED, Goodison S, et al. IL-8 as a urinary biomarker for the detection of bladder cancer. BMC Urol. 2012;12:12.

45. Kamat AM, Briggman J, Urbauer DL, Svatek R, Nogueras González GM, Anderson R, et al. Cytokine Panel for Response to Intravesical Therapy (CyPRIT): Nomogram of Changes in Urinary Cytokine Levels Predicts Patient Response to Bacillus Calmette-Guérin. Eur Urol. 2016;69(2):197-200.

46. Kumari N, Agrawal U, Mishra AK, Kumar A, Vasudeva P, Mohanty NK, et al. Predictive role of serum and urinary cytokines in invasion and recurrence of bladder cancer. Tumour Biol. 2017;39(4):1010428317697552.

47. Reis ST, Leite KR, Piovesan LF, Pontes-Junior J, Viana NI, Abe DK, et al. Increased expression of MMP-9 and IL-8 are correlated with poor prognosis of Bladder Cancer. BMC Urol. 2012;12:18.

48. Koçak H, Oner-Iyidoğan Y, Koçak T, Oner P. Determination of diagnostic and prognostic values of urinary interleukin-8, tumor necrosis factor-alpha, and leukocyte arylsulfatase-A activity in patients with bladder cancer. Clin Biochem. 2004;37(8):673-8.

49. Sheryka E, Wheeler MA, Hausladen DA, Weiss RM. Urinary interleukin-8 levels are elevated in subjects with transitional cell carcinoma. Urology. 2003;62(1):162-6.

50. Welinder C, Jirström K, Lehn S, Nodin B, Marko-Varga G, Blixt O, et al. Intra-tumour IgA1 is common in cancer and is correlated with poor prognosis in bladder cancer. Heliyon. 2016;2(8):e00143.

51. Nutt JE, Mellon JK, Qureshi K, Lunec J. Matrix metalloproteinase-1 is induced by epidermal growth factor in human bladder tumour cell lines and is detectable in urine of patients with bladder tumours. Br J Cancer. 1998;78(2):215-20.

52. Durkan GC, Nutt JE, Rajjayabun PH, Neal DE, Lunec J, Mellon JK. Prognostic significance of matrix metalloproteinase-1 and tissue inhibitor of metalloproteinase-1 in voided urine samples from patients with transitional cell carcinoma of the bladder. Clin Cancer Res. 2001;7(11):3450-6.

53. Zoidakis J, Makridakis M, Zerefos PG, Bitsika V, Esteban S, Frantzi M, et al. Profilin 1 is a potential biomarker for bladder cancer aggressiveness. Mol Cell Proteomics. 2012;11(4):M111.009449.

54. Vikey A. D-dimer as an alarming biomarker in various cancers: A review of literature. 2018;1.

55. Kang HW, Kim W-J, Yun S-J. The role of the tumor microenvironment in bladder cancer development and progression - Kang - Translational Cancer Research. 2022.

56. Waugh DJ, Wilson C. The interleukin-8 pathway in cancer. Clin Cancer Res. 2008;14(21):6735-41.

57. Miao C, Liang C, Zhu J, Xu A, Zhao K, Hua Y, et al. Prognostic role of matrix metalloproteinases in bladder carcinoma: a systematic review and meta-analysis. Oncotarget. 2017;8(19):32309-21.

**References solely in this “Additional file”**

58. Chen X, Ji H, Wang J, Zhao G, Zheng B, Niu Z, et al. Prognostic Value of the Preoperative Plasma D-Dimer Levels in Patients with Upper Tract Urothelial Carcinoma in a Retrospective Cohort Study. Onco Targets Ther. 2020;13:5047-55.

59. Zareba P, Duivenvoorden WCM, Pinthus JH. Thromboembolism in Patients with Bladder Cancer: Incidence, Risk Factors and Prevention. Bladder Cancer. 2018;4(2):139-47.

60. Salem H, Ellakwa DE-S, Fouad H, Hamid MA. APOA1 AND APOA2 proteins as prognostic markers for early detection of urinary bladder cancer. Gene Reports. 2019;16:100463.

61. Chen YT, Chen CL, Chen HW, Chung T, Wu CC, Chen CD, et al. Discovery of novel bladder cancer biomarkers by comparative urine proteomics using iTRAQ technology. J Proteome Res. 2010;9(11):5803-15.

62. Li CY, Li HJ, Zhang T, Gao HS, Chang JW, Men XL, et al. [Significance of apolipoprotein A1 as biomarker for early diagnosis and classification of bladder urothelial carcinoma]. Zhonghua Lao Dong Wei Sheng Zhi Ye Bing Za Zhi. 2013;31(4):266-70.

63. Halder S, Dey RK, Chowdhury AR, Bhattacharyya P, Chakrabarti A. Differential regulation of urine proteins in urothelial neoplasm. J Proteomics. 2015;127(Pt A):185-92.

64. Nedjadi T, Albarakati N, Benabdelkamel H, Masood A, Alfadda AA, Al-Maghrabi J. Proteomic Profiling of Plasma-Derived Biomarkers in Patients with Bladder Cancer: A Step towards Clinical Translation. Life (Basel). 2021;11(12).

65. Ren L, Yi J, Li W, Zheng X, Liu J, Wang J, et al. Apolipoproteins and cancer. Cancer Med. 2019;8(16):7032-43.

66. Yao R, Davidson DD, Lopez-Beltran A, MacLennan GT, Montironi R, Cheng L. The S100 proteins for screening and prognostic grading of bladder cancer. Histol Histopathol. 2007;22(9):1025-32.

67. Dokun OY, Florl AR, Seifert HH, Wolff I, Schulz WA. Relationship of SNCG, S100A4, S100A9 and LCN2 gene expression and DNA methylation in bladder cancer. Int J Cancer. 2008;123(12):2798-807.

68. Hegele A, Heidenreich A, Varga Z, von Knobloch R, Olbert P, Kropf J, et al. Cellular fibronectin in patients with transitional cell carcinoma of the bladder. Urol Res. 2003;30(6):363-6.

69. Habash NK, Abdul-Rasheed OF, Al-Nasiri US. Evaluation of serum and urinary fibronectin as a diagnostic marker of bladder cancer. 2014;12:267-72.

70. Malmström PU, Larsson A, Johansson S. Urinary fibronectin in diagnosis and follow-up of patients with urinary bladder cancer. Br J Urol. 1993;72(3):307-10.

71. Inoue K, Slaton JW, Kim SJ, Perrotte P, Eve BY, Bar-Eli M, et al. Interleukin 8 expression regulates tumorigenicity and metastasis in human bladder cancer. Cancer Res. 2000;60(8):2290-9.

72. Margel D, Pevsner-Fischer M, Pesvner-Fischer M, Baniel J, Yossepowitch O, Cohen IR. Stress proteins and cytokines are urinary biomarkers for diagnosis and staging of bladder cancer. Eur Urol. 2011;59(1):113-9.

73. Betkerur V, Rao R, Hlaing V, Rhee H, Baumgartner G, Guinan P. Screening tests for detection of bladder cancer. Urology. 1980;16(1):16-9.

74. Rodriguez Faba O, Palou-Redorta J, Fernández-Gómez JM, Algaba F, Eiró N, Villavicencio H, et al. Matrix Metalloproteinases and Bladder Cancer: What is New? ISRN urology. 2012;2012:581539-.

75. Wallard MJ, Pennington CJ, Veerakumarasivam A, Burtt G, Mills IG, Warren A, et al. Comprehensive profiling and localisation of the matrix metalloproteinases in urothelial carcinoma. Br J Cancer. 2006;94(4):569-77.

76. Ishchuk TV, Glavachek DO, Savchuk OM, Yakovlev PG, Falaleeva TM, Beregova TV, et al. Plasma levels of MMPs and TIMP-1 in urinary bladder cancer patients. *Biomedical Research and Therapy*. 2018;5(1):1931-40.

77. Offersen BV, Knap MM, Horsman MR, Verheijen J, Hanemaaijer R, Overgaard J. Matrix metalloproteinase-9 measured in urine from bladder cancer patients is an independent prognostic marker of poor survival. Acta Oncol. 2010;49(8):1283-7.

78. Zeng FC, Cen S, Tang ZY, Kang XL. Elevated matrix metalloproteinase-9 expression may contribute to the pathogenesis of bladder cancer. Oncol Lett. 2016;11(3):2213-22.

79. Wu GJ, Bao JS, Yue ZJ, Zeng FC, Cen S, Tang ZY, et al. Elevated expression of matrix metalloproteinase-9 is associated with bladder cancer pathogenesis. J Cancer Res Ther. 2018;14(Supplement):S54-S9.

80. Chuang CK, Pang ST, Chuang TJ, Liao SK. Profiling of matrix metalloproteinases and tissue inhibitors of metalloproteinases proteins in bladder urothelial carcinoma. Oncol Lett. 2010;1(4):691-5.

81. El-Sharkawi F, El Sabah M, Hassan Z, Khaled H. The biochemical value of urinary metalloproteinases 3 and 9 in diagnosis and prognosis of bladder cancer in Egypt. J Biomed Sci. 2014;21:72.

82. Gerhards S, Jung K, Koenig F, Daniltchenko D, Hauptmann S, Schnorr D, et al. Excretion of matrix metalloproteinases 2 and 9 in urine is associated with a high stage and grade of bladder carcinoma. Urology. 2001;57(4):675-9.

83. Kouser L, Abdul-Aziz M, Nayak A, Stover CM, Sim RB, Kishore U. Properdin and factor h: opposing players on the alternative complement pathway "see-saw". Front Immunol. 2013;4:93.

84. Lemańska-Perek A, Lis-Kuberka J, Lepczyński A, Dratwa-Chałupnik A, Tupikowski K, Kątnik-Prastowska I, et al. Potential plasma biomarkers of bladder cancer identified by proteomic analysis: A pilot study. Adv Clin Exp Med. 2019;28(3):339-46.

85. Dmytryk V, Luhovska T, Yakovlev P, Savchuk O, Halenova T, Raksha N, et al. Proteolytic parameter changes in the plasma of patients with bladder cancer – depending on tumor stage. Current Issues in Pharmacy and Medical Sciences. 2020;33(2):67-71.

86. Shabayek MI, Sayed OM, Attaia HA, Awida HA, Abozeed H. Diagnostic evaluation of urinary angiogenin (ANG) and clusterin (CLU) as biomarker for bladder cancer. Pathol Oncol Res. 2014;20(4):859-66.

87. Hazzaa SM, Elashry OM, Afifi IK. Clusterin as a diagnostic and prognostic marker for transitional cell carcinoma of the bladder. Pathol Oncol Res. 2010;16(1):101-9.

88. Ekici S, Eroğlu A, Doğan Ekici AI, Türkeri L. Clusterin immunoreactivity as a predictive factor for progression of non-muscle-invasive bladder carcinoma. Urol Int. 2011;86(1):31-5.

89. M P, J D, S Z, T T, Q S, X Y, et al. The role of Clusterin in cancer metastasis. Cancer management and research. 2019;11.

90. Alfano M, Canducci F, Nebuloni M, Clementi M, Montorsi F, Salonia A. The interplay of extracellular matrix and microbiome in urothelial bladder cancer. Nat Rev Urol. 2016;13(2):77-90.

91. Roudnicky F, Poyet C, Wild P, Krampitz S, Negrini F, Huggenberger R, et al. Endocan is upregulated on tumor vessels in invasive bladder cancer where it mediates VEGF-A-induced angiogenesis. Cancer Res. 2013;73(3):1097-106.

92. Laloglu E, Aksoy H, Aksoy Y, Ozkaya F, Akcay F. The determination of serum and urinary endocan concentrations in patients with bladder cancer. Ann Clin Biochem. 2016;53(6):647-53.

93. Huang X, Chen C, Wang X, Zhang JY, Ren BH, Ma DW, et al. Prognostic value of endocan expression in cancers: evidence from meta-analysis. Onco Targets Ther. 2016;9:6297-304.

94. Shi C, Sun L, Liu S, Zhang E, Song Y. Overexpression of Karyopherin Subunit alpha 2 (KPNA2) Predicts Unfavorable Prognosis and Promotes Bladder Cancer Tumorigenicity via the P53 Pathway. Med Sci Monit. 2020;26:e921087.

95. Xu C, Liu M. Integrative bioinformatics analysis of KPNA2 in six major human cancers. Open Med (Wars). 2021;16(1):498-511.

96. Zeng F, Luo L, Li D, Guo J, Guo M. KPNA2 interaction with CBX8 contributes to the development and progression of bladder cancer by mediating the PRDM1/c-FOS pathway. J Transl Med. 2021;19(1):112.

97. JB J, PP M, CM S, N F, K B-D, BP U, et al. High expression of karyopherin-α2 defines poor prognosis in non-muscle-invasive bladder cancer and in patients with invasive bladder cancer undergoing radical cystectomy. European urology. 2011;59(5).

98. J Z, D D, R C, Y W, S J, Y Z, et al. Aberrant expression of KPNA2 is associated with a poor prognosis and contributes to OCT4 nuclear transportation in bladder cancer. Oncotarget. 2016;7(45).

99. Tang T, Yang ZY, Wang D, Yang XY, Wang J, Li L, et al. The role of lysosomes in cancer development and progression. Cell Biosci. 2020;10(1):131.

100. Wieczorek E, Jablonowski Z, Tomasik B, Gromadzinska J, Jablonska E, Konecki T, et al. Different Gene Expression and Activity Pattern of Antioxidant Enzymes in Bladder Cancer. Anticancer Res. 2017;37(2):841-8.

101. Antonova O, Rukova B, Mladenov B, Rangelov S, Hammoudeh Z, Nesheva D, et al. Expression profiling of muscle invasive and non-invasive bladder tumors for biomarkers identification related to drug resistance, sensitivity and tumor progression. *Biotechnology & Biotechnological Equipment*. 2020;34:506–14.

102. Kader AK, Liu J, Shao L, Dinney CP, Lin J, Wang Y, et al. Matrix metalloproteinase polymorphisms are associated with bladder cancer invasiveness. Clin Cancer Res. 2007;13(9):2614-20.
